# Computational Modeling of Hybrid Carbon Fiber/Epoxy Composites Reinforced with Functionalized and Non-Functionalized Graphene Nanoplatelets

**DOI:** 10.3390/nano11112919

**Published:** 2021-10-31

**Authors:** Hashim Al Mahmud, Matthew S. Radue, William A. Pisani, Gregory M. Odegard

**Affiliations:** 1 Department of Mechanical Engineering, University of Kufa, P.O. Box 21, Kufa 54003, Iraq; hashimn.almahmood@uokufa.edu.iq or; 2 Department of Mechanical Engineering-Engineering Mechanics, Michigan Technological University, Houghton, MI 49931, USA; msradue@mtu.edu; 3 Oak Ridge Institute for Science and Education, Oak Ridge, TN 37830, USA; william.a.pisani@usace.army.mil; 4 Environmental Laboratory, US Army Engineer Research and Development Center, Vicksburg, MS 39180, USA

**Keywords:** composite laminate, functionalization, nanoplatelet content, nanoplatelet aspect ratio, micromechanics

## Abstract

The mechanical properties of aerospace carbon fiber/graphene nanoplatelet/epoxy hybrid composites reinforced with pristine graphene nanoplatelets (GNP), highly concentrated graphene oxide (GO), and Functionalized Graphene Oxide (FGO) are investigated in this study. By utilizing molecular dynamics data from the literature, the bulk-level mechanical properties of hybrid composites are predicted using micromechanics techniques for different graphene nanoplatelet types, nanoplatelet volume fractions, nanoplatelet aspect ratios, carbon fiber volume fractions, and laminate lay-ups (unidirectional, cross-ply, and angle-ply). For the unidirectional hybrid composites, the results indicate that the shear and transverse properties are significantly affected by the nanoplatelet type, loading and aspect ratio. For the cross-ply and angle ply hybrid laminates, the effect of the nanoplate’s parameters on the mechanical properties is minimal when using volume fractions and aspect ratios that are typically used experimentally. The results of this study can be used in the design of hybrid composites to tailor specific laminate properties by adjusting nanoplatelet parameters.

## 1. Introduction

The development of the next generation of high-performance composite materials has been motivated by increasing performance demands in applications such as aerospace and wind energy. One route to producing composites with improved properties over state-of-the-art systems involves the use of nanoparticle reinforcement inside traditional fiber-reinforced thermosetting resins. Nanoparticles such as carbon nanotubes and graphene have extraordinary mechanical properties, and thus these hybrid nanoparticle and fiber-reinforced composites can potentially achieve greater stiffness, strength, and toughness relative to traditional fiber-reinforced composites [1,2,3,4,5,6,7,8,9,10,11]. However, the development of nanoparticle hybrid composites is still in its infancy.

A significant number of studies have addressed the fabrication, characterization, and testing of hybrid composite materials [2,12,13,14,15,16,17,18,19,20,21,22,23,24]. Although some preliminary success has been achieved in terms of obtaining mechanical properties that exceed those of traditional fiber composites, there are numerous variables in hybrid composite material design (e.g., fiber layer, fiber volume fraction, nanoparticle volume fraction, nanoparticle surface treatment). The traditional trial-and-error experimental design process of hybrid composites is prohibitively expensive, preventing the proper searching of the design space for high-performance systems. Fortunately, computationally driven material design is an efficient, accurate, and overall more optimal approach for developing novel, high-performance systems.

There are a few multiscale computational options for developing novel, high-performance hybrid composites. The finite element method (FEM) is a powerful technique capable of the multiscale modeling of hybrid polymer composites. A wide variety of variables can be investigated when using FEM to model polymer composites, including dispersion, waviness, agglomeration, and nanoparticle loading [25]. It is also possible to investigate the interfacial shear strength of the fiber and matrix using FEM [26]. Complex geometries, such as radially aligned carbon nanotube-coated carbon fibers in a polymer matrix, and automotive drive shafts, can be simulated [26,27].

Another computational option for understanding the structure–property relationships of hybrid composites is a multi-scale approach comprising molecular dynamics (MD) simulation and micromechanics theory. MD simulation is an accurate and efficient computational method for predicting the mechanical properties of polymers and their composites [1,2,28,29,30,31,32,33,34]. Unlike FEM, MD simulation can capture molecular-level phenomena that significantly influence the mechanical response at the macro-scale. Additionally, MD simulation can help to understand interfacial interactions (i.e., load transfer) between the polymer matrix and nanoparticle reinforcement. While MD simulation can predict the mechanical properties of polymer composites at the nanometer scale, it cannot predict the mechanical properties of laminate-level hybrid composites. Among several approaches for micromechanics analysis, NASA’s Glenn Research Center’s Micromechanics Analysis Code based on the Generalized Method of Cells (MAC/GMC) is an accurate, efficient, and validated micromechanics tool that can be used to predict laminate-level mechanical properties [2,35,36,37,38]. The potential of this micromechanics tool has been exploited in many computational studies to develop composite structures [1,2,3,4,11,30,39,40]. 

The interaction between the nanoparticle and polymer matrix dictates the level of reinforcement load transfer, and therefore significantly affects the composite’s mechanical properties. The nanoparticle–polymer interface can be strengthened by functionalizing the nanoparticle [41]. Functionalization consists of incorporating functional groups such as amine (-NH2), amide (-O=C-NH2), graphitic nitrogen (-N-), hydroxyl (-OH), and epoxide (-O-) into the nanoparticle. While still difficult to achieve experimentally, nanoparticles can be functionalized easily and precisely through MD simulation approaches [42,43,44,45]. Al Mahmud et al. [42] developed an experimentally validated multi-scale modeling approach for predicting the mechanical properties of graphene nanoplatelet/epoxy composites for different types of chemical functionalization of graphene nanoplatelets. However, their analysis did not extend to carbon fiber (CF)/nanoplatelet/epoxy hybrid composites. To enable the rapid design of hybrid composites with nanoplatelet functionalization for specific structural applications, a more comprehensive set of predictions is necessary to relate material structure to structural performance. 

The objective of this research is to utilize multiscale modeling to determine the structural behavior of CF/graphene nanoplatelet/epoxy hybrid composite laminates for different levels of nanoplatelet functionalization, aspect ratio, and loading, as well as CF loading and layup. The monograph is intended to produce a useful database for experimentalists and/or manufacturers, which will help to reduce the time, cost, and effort involved in developing these composite materials. Such comprehensive data are not currently available in the literature for this material and level of accuracy. The results provide predictions of the laminate-level response of hybrid composites based on nanoscopic, microscopic, and macroscopic material parameters. First, the modeling methods will be described, followed by a comprehensive discussion of the results.

## 2. Materials and Methods

The details of the simulated material and the modeling procedures are presented in this section. The modeling described herein builds off of two multiscale modeling studies: one on dispersed and agglomerated CF/graphene nanoplatelet/epoxy hybrid composites [1], and another on functionalized and non-functionalized graphene nanoplatelet/epoxy nanocomposites [42]. In the current study, the results from both of these studies are utilized in a micromechanics framework to predict the laminate-level properties of hybrid composites with a wide range of material design parameters, including dispersion, functionalization chemistry, aspect ratio, and volume fraction. The simulated epoxy system is a DGEBA/F (diglycidyl ether of bisphenol F/A) monomer crosslinked with DETDA (diethyltoluenediamine). The details of the epoxy system and the simulation protocols for all the MD modeling results used in this work can be found elsewhere [1,42]. For all micromechanical simulations described herein, the aforementioned MAC/GMC code was used. 

MAC/GMC is a comprehensive, user-friendly, and efficient computer code developed at NASA’s Glenn Research Center. The computational approach in this code is based on the High-Fidelity Generalized Method of Cells (HFGMC) micromechanics theory. HFGMC is more accurate and efficient in predicting the local stress and strain fields relative to the standard GMC. This improvement is essential to providing accurate predictions and more detailed analyses of composite materials. Specifically, the availability of accurate local stress and strain fields is critical for accurately modeling the interphase region between composite constituents. This level of accuracy is necessary to analyze composite material damage and failures, such as matrix inelasticity and fiber–matrix debonding. Via this micromechanics approach, the microscale architecture of constituents was characterized using a doubly and triply periodic repeating unit cell (RUC) to model the mechanical behavior and response. The RUC is composed of a number of subcells. Each subcell can be used to represent a single phase of heterogeneous or composite materials. Thus, the code can be used to analyze a wide range of material constitutive models. The constitutive model of the material could be isotropic, transversely isotropic, or completely anisotropic. 

### 2.1. Nanoplatelets

Studies on polymer-based nanocomposites reinforced with graphene nanoplatelets have referred to a critical problem, represented by the difficulty of preserving the perfect dispersion of the graphene nanoplatelets within the polymer matrix. This is because graphene nanoplatelets have a natural tendency to agglomerate and form particles of stacked graphene layers within the polymer matrix. This problem is referred to as the agglomeration phenomenon, and is triggered by the noncovalent Van der Waals forces and pi-conjugation. It is an unfavorable phenomenon because it impedes the dispersion of graphene nanoplatelets in polymer matrices [46,47,48,49,50,51]. Therefore, significant degradation in the reinforcing function of graphene nanoplatelets is commonly observed at poor dispersion levels. This is because of the decrease in the interfacial contact surface area between the nanoplatelets and the matrix, in addition to the formation of easy slip planes within the reinforcement particles [50,51]. To prevent the agglomeration phenomenon, different mixing and stirring techniques have been adopted in the process of preparing polymer-based nanocomposites, with the aid of adding liquid solvents and sonication to maintain better dispersion. Additionally, the chemical modification of graphene nanolayers (functionalization) has been commonly utilized for improving their dispersion level and to enhance the interfacial covalent bonding with polymer matrices [52,53,54]. 

**Figure 1:**
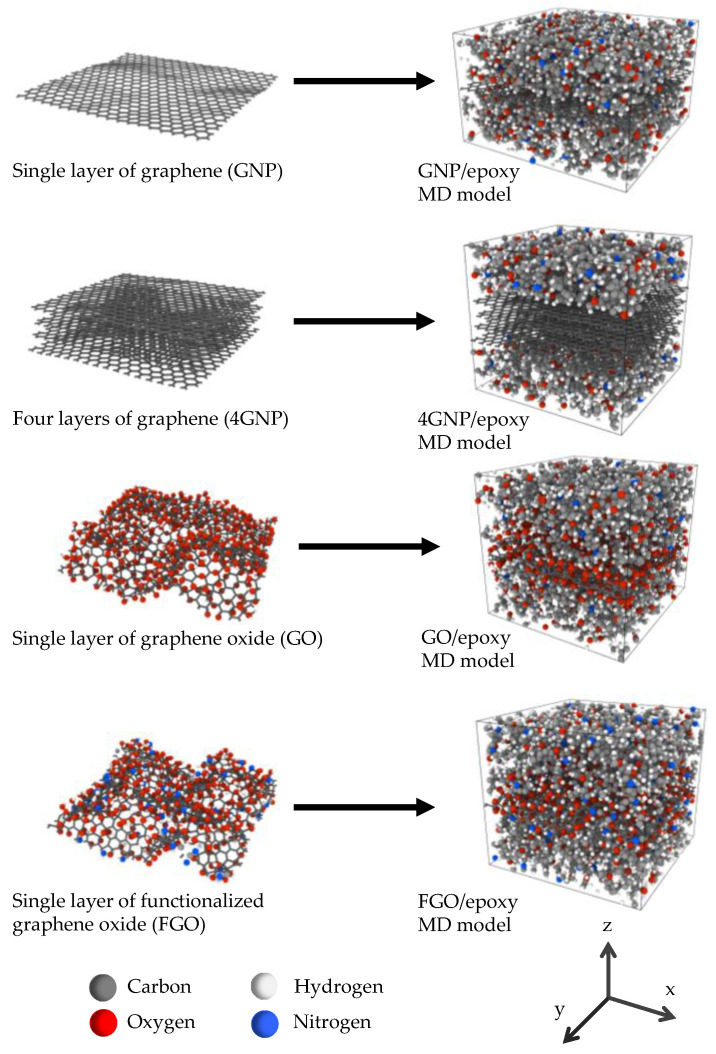
Representative MD models of GNP, 4GNP, GO, and FGO nanoplatelets and their corresponding nanoplatelet/epoxy MD models.

Given the discussion mentioned above, four different graphene nanoplatelet types were considered in this study: pristine well-dispersed graphene nanoparticles (GNP), agglomerated nanoparticles (4GNP), graphene nanoparticles functionalized with oxygen groups (GO), and graphene nanoparticles functionalized with oxygen and amine groups (FGO). Molecular dynamics (MD) images of these four nanoplatelet types and the corresponding nanoplatelet/epoxy MD models are shown in Figure 1. It was shown by Al Mahmud et al. [42] that the complete dispersion of graphene nanoplatelets corresponds to improved mechanical properties relative to poor dispersion. The purpose of chemical functionalization is to increase the load transfer capability of the nanoparticle/epoxy interface. As demonstrated by Al Mahmud et al. [42], excessive functionalization increases the relative interaction energy of the interface and the waviness of the nanoplatelets, but degrades the robustness of the nanoplatelets, and thus the overall Young’s and shear moduli of the nanocomposite. 

### 2.2. Nanoplatelet/Epoxy Composite Modeling

**Figure 2:**
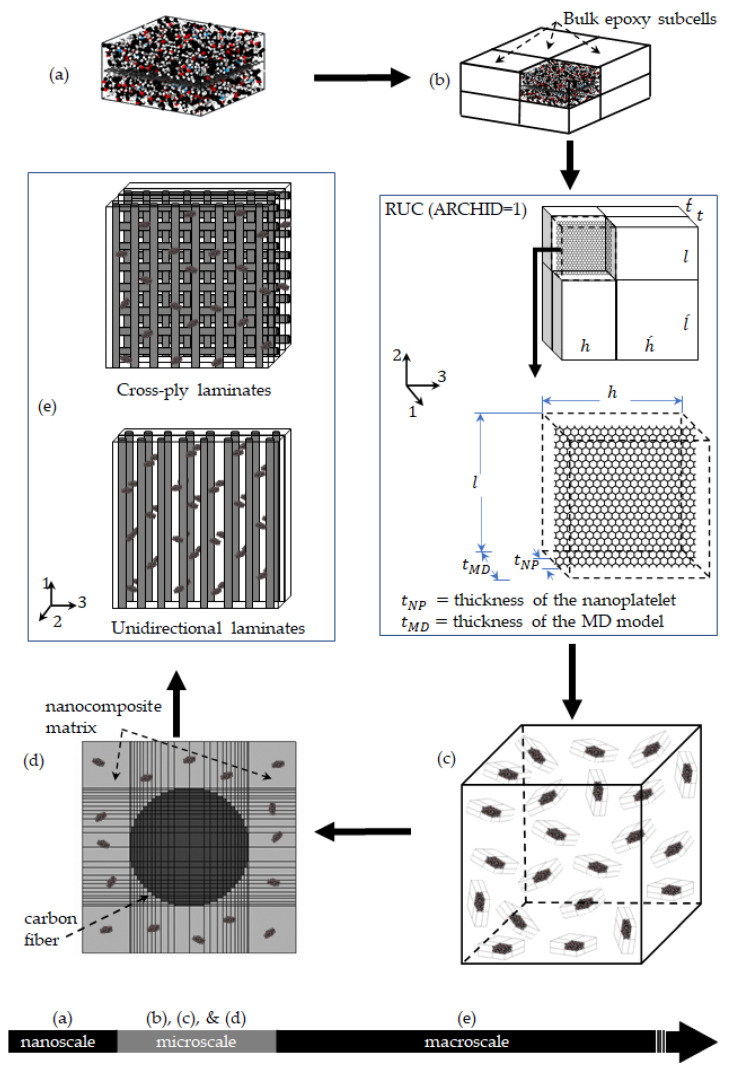
The multiscale modeling workflow; (**a**) MD model: Local interphase region of nanoplatelet/epoxy, (**b**) MAC/GMC RUC ARCHID = 1, (**c**) bulk nanocomposite: randomly-oriented nanoplatelets within the epoxy matrix, (**d**) MAC/GMC RUC (ARCHID = 13) to generate unidirectional CF/nanoplatelets/epoxy hybrid composite laminate, (**e**) hybrid composite laminates.

Al Mahmud et al. [1,42] used a multiscale modeling method to predict the elastic properties of graphene nanoplatelet/epoxy nanocomposites with the GNP, 4GNP, GO, and FGO nanoplatelet types. Molecular dynamics (MD) simulation was used to predict the mechanical response of the nanoplatelet/epoxy interfacial area at the nanoscale, and micromechanics was used to subsequently determine the mechanical response of epoxy reinforced with various levels and aspect ratios of randomly oriented nanoplatelets. A schematic of this modeling approach is shown in Figure 2. Figure 2a shows the MD model of the interfacial region, which incorporates the effects of graphene nanoplatelet agglomeration and functionalization, as well as the disturbance of the polymer’s molecular structure at the interface with the nanoplatelet. The overall elastic properties of the MD simulation box are provided in Table 1, where the in-plane properties correspond to the x–y plane (Figure 1), and the out-of-plane properties correspond to the z-axis. Further details of the MD simulation can be found elsewhere [1,42].

Figure 2b,c illustrate the micromechanical modeling steps toward establishing the nanoplatelet/epoxy properties. Figure 2b illustrates the incorporation of the MD predicted mechanical properties into a micromechanical continuum model, which includes regions of bulk epoxy properties, the size of which can be adjusted to control the simulated nanoplatelet volume fraction. This step predicts the properties of an aligned graphene/epoxy composite. Figure 2c shows the following step, in which the aligned nanoplatelet/epoxy properties are used to predict the resulting properties of an epoxy system reinforced with randomly oriented nanoplatelets. Further details on this stage of micromechanical modeling can be found elsewhere [1,42].

### 2.3. Micromechanics of CF/Nanoplatelet/Epoxy Hybrid Composites

For the current study, using the nanoplatelet/epoxy homogenized mechanical properties that were predicted as described above, a second MAC/GMC script was developed to simulate the CF/nanoplatelet/epoxy hybrid composite systems. Figure 2d shows the built-in RUC of a 26 × 26 circular array to represent the CF architecture within the nanocomposite matrix. For comparison reasons, the AS4 CF, which was used in previous multiscale modeling studies [1,2,31], was modeled herein to reinforce the nanocomposite matrix. The assumed axial, transverse, and shear moduli of the AS4 CF were 231, 9.6, and 112 GPa, respectively. The Poisson’s ratio was 0.3, and a 56% CF volume fraction was assumed for all micromechanics calculations. One ply of a unidirectional CF/nanoplatelet/epoxy hybrid composite and three different arrangements of laminated hybrid composites were modeled in this work. Figure 2e shows representative example sketches of the unidirectional and cross-ply laminate hybrid composites.

## 3. Results and Discussions

The predicted mechanical properties of the CF/nanoplatelet/epoxy hybrid composites are described in this section. The predictions for the unidirectional plies are presented first, followed by the laminates.

### 3.1. Unidirectional CF-Based Hybrid Composites

**Figure 3:**
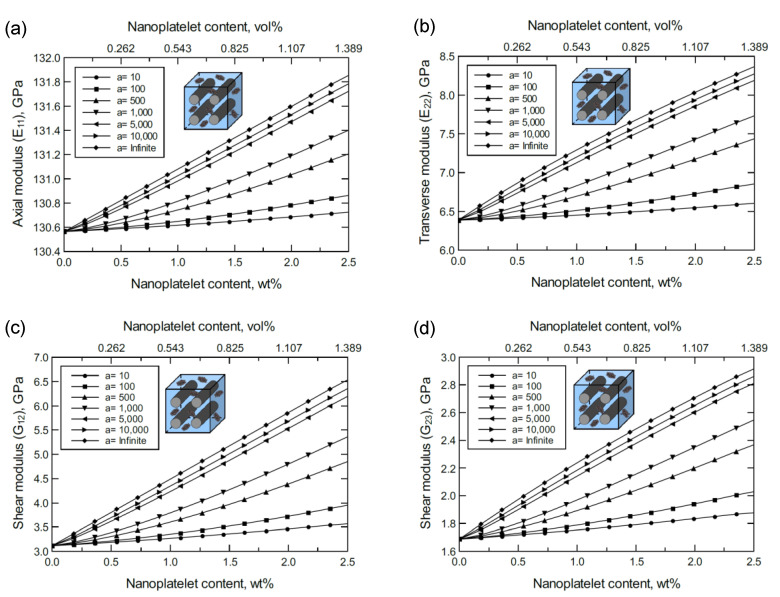
Predicted elastic and shear moduli for unidirectional CF/GNP/epoxy for various nanoplatelet content and aspect ratio values; (**a**) axial elastic modulus $E_{11}$, (**b**) transverse elastic modulus $E_{22}$, (**c**) shear modulus $G_{12}$, (**d**) shear modulus $G_{23}$. The volume fraction of CF is 56%.

The simulation for the unidirectional CF-based hybrid composites was performed as illustrated in Figure 2d,e. Figure 3 shows the predicted axial ($E_{11}$), transverse ($E_{22}=E_{33}$), and shear ($G_{12}$*,* $G_{23}$) moduli of the unidirectional CF/GNP/Epoxy hybrid composite. Generally, there is an improvement in the mechanical response as the GNP content and/or aspect ratio increase. Referring to the nanoplatelet configuration and details shown in Figure 2b, the aspect ratio was assumed as the ratio of the nanoplatelet length to its thickness ($a=l/t_{NP}$). The effectiveness of the reinforcement, however, varies according to the direction or plane of the measured mechanical property. 

**Figure 4:**
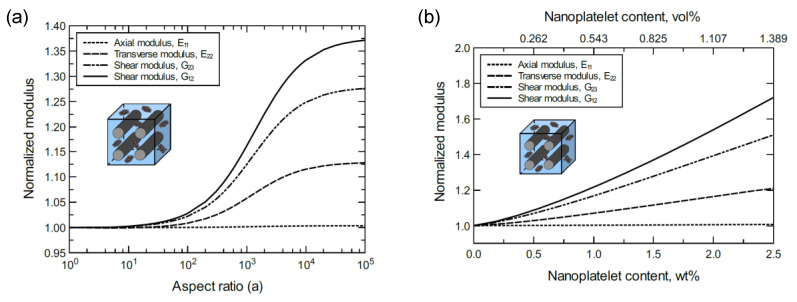
Normalized elastic and shear moduli for unidirectional CF/GNP/epoxy (**a**) at 1.0 wt% of GNP content and various GNP aspect ratios, (**b**) at 10^3^ GNP aspect ratio and various GNP contents. The volume fraction of CF is 56%.

To better discern the reinforcing effect of the GNP on each mechanical property of the hybrid composite, the mechanical properties were normalized and plotted together in one graph. For the reinforcing effect caused by increasing the GNP aspect ratio, Figure 4a shows the normalized $E_{11}$, $E_{22}$, $G_{23}$ and $G_{12}$ predicted at 1.0 wt% of GNP. The reinforcing effect caused by increasing the GNP content is shown in Figure 4b for a 10^3^ GNP aspect ratio. Clearly, the greatest improvement can be observed in the shear moduli with $G_{12}$ > $G_{23}$. Conversely, $E_{11}$ involves a trivial improvement relative to $E_{22}$ due to the CF domination along the axial direction. As a result, the overall order of the reinforcing effect on the mechanical properties is $G_{12}$ > $G_{23}$ > $E_{22}$ > $E_{11}$. The obtained results imply that the material rigidity of the unidirectional CF composite laminate is highly sensitive to GNP inclusion. While little improvement in $E_{11}$ is expected because of the presence of stiff carbon fibers, the large improvement in shear is generally due to the increase in interfacial load transfer. This is especially relevant for longitudinal shear ($G_{12}$), where matrix/reinforcement load transfer has a significant impact on composite elastic properties.

**Figure 5:**
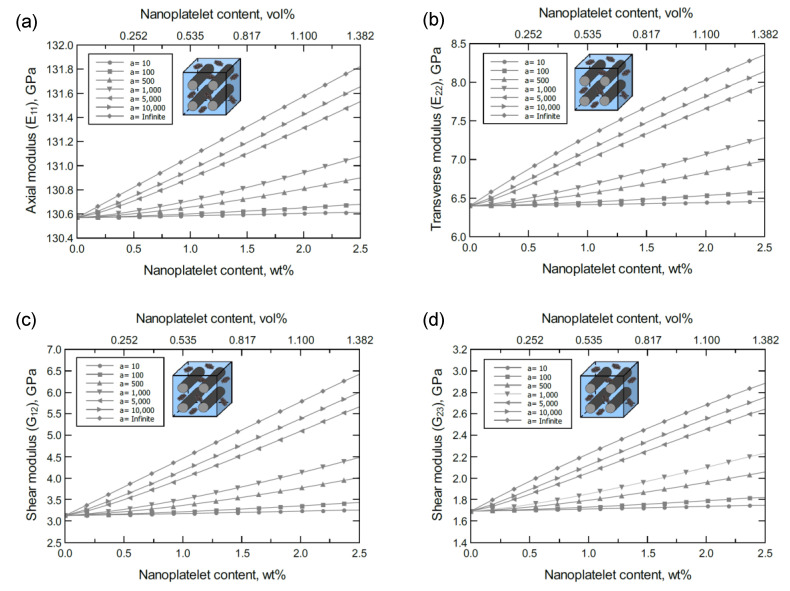
Predicted elastic and shear moduli for a unidirectional CF/4GNP/epoxy for various nanoplatelet content and aspect ratio values; (**a**) axial elastic modulus $E_{11}$, (**b**) transverse elastic modulus $E_{22}$, (**c**) shear modulus $G_{12}$, (**d**) shear modulus $G_{23}$. The volume fraction of CF is 56%.

**Figure 6:**
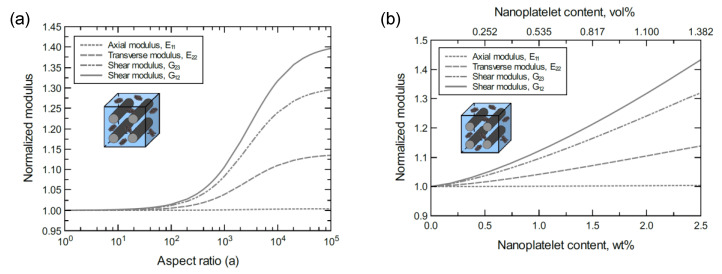
Normalized elastic and shear moduli for unidirectional CF/4GNP/epoxy (**a**) at 1.0 wt% of 4GNP and various 4GNP aspect ratios, (**b**) at 10^3^ 4GNP aspect ratio and various 4GNP contents. The volume fraction of CF is 56%.

At lower levels of GNP dispersion, the mechanical responses of unidirectional CF/4GNP/epoxy hybrid composites exhibit a similar trend to that observed for the unidirectional CF/GNP/epoxy. Figure 5 shows an improvement in the predicted $E_{11}$, $E_{22}$, $G_{12}$, and $G_{23}$ moduli of the unidirectional CF/4GNP/epoxy hybrid composite as either the 4GNP content or aspect ratio increases. The normalized mechanical properties predicted for 1.0 wt% of 4GNP content and various aspect ratios are shown in Figure 6a. Figure 6b shows the normalized mechanical properties predicted at a 10^3^ aspect ratio for various values of 4GNP content. Once again, the reinforcing effects of the 4GNP on $G_{12}$ surpasses that of $G_{23}$, while both exceed the reinforcing effect observed for $E_{22}$. An insignificant improvement is also observed in $E_{11}$ as it is dominated by the CF.

**Figure 7:**
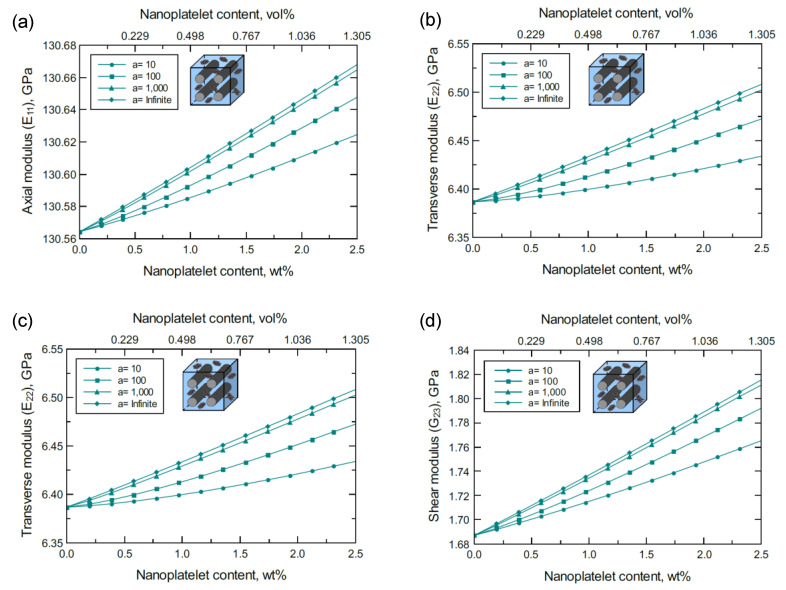
Predicted elastic and shear moduli for unidirectional CF/GO/epoxy for various nanoplatelet content and aspect ratio values; (**a**) axial elastic modulus $E_{11}$, (**b**) transverse elastic modulus $E_{22}$, (**c**) shear modulus $G_{12}$, (**d**) shear modulus $G_{23}$. The volume fraction of CF is 56%.

**Figure 8:**
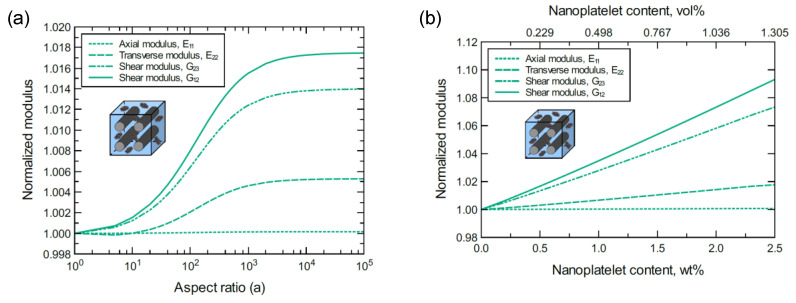
Normalized elastic and shear moduli for unidirectional CF/GO/epoxy (**a**) at 1.0 wt% GO content and various GO aspect ratios, (**b**) at 10^3^ GO aspect ratio and various GO contents. The volume fraction of CF is 56%.

**Figure 9:**
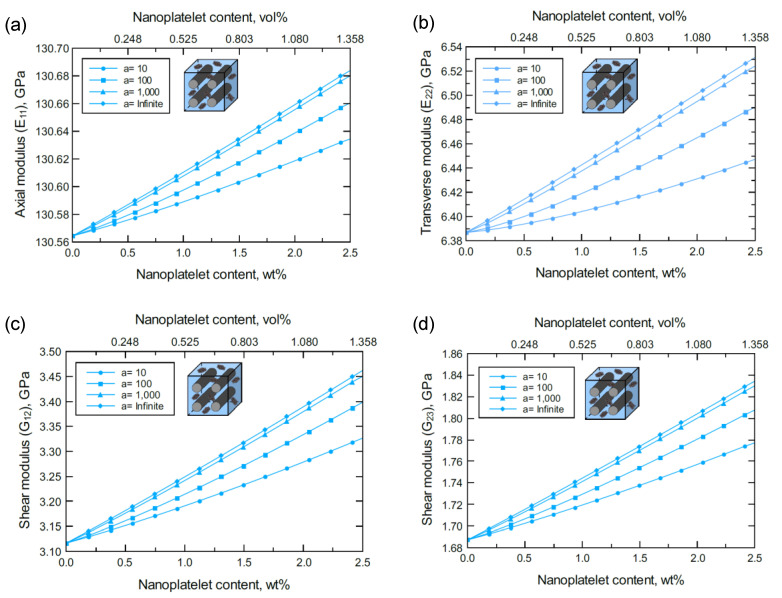
Predicted elastic and shear moduli for unidirectional CF/FGO/epoxy for various nanoplatelet content and aspect ratio values; (**a**) axial elastic modulus $E_{11}$, (**b**) transverse elastic modulus $E_{22}$, (**c**) shear modulus $G_{12}$, (**d**) shear modulus $G_{23}$. The volume fraction of CF is 56%.

**Figure 10:**
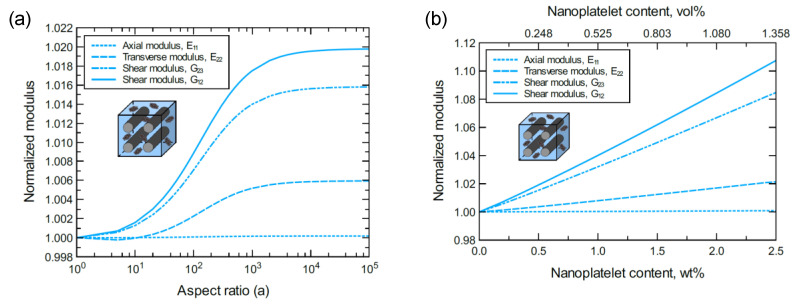
Normalized elastic and shear moduli for unidirectional CF/FGO/epoxy (**a**) at 1.0 wt% FGO content and various FGO aspect ratios, (**b**) at 10^3^ FGO aspect ratio and various FGO contents. The volume fraction of CF is 56%.

Figure 7, Figure 8, Figure 9 and Figure 10 show the mechanical response of unidirectional CF/GO/epoxy and unidirectional CF/FGO/epoxy hybrid composites. Even though the FGO-reinforced hybrid composite demonstrates a slightly better mechanical response relative to the GO-reinforced hybrid composite, both hybrid composites show a limited improvement in the mechanical properties with increases in the nanoplatelet content and/or aspect ratio. The range of improvement for the hybrid composite reinforced with GO or FGO is very small in comparison to the hybrid composite reinforced with GNP or 4GNP. However, a similar behavior can be observed when comparing the reinforcing effect on each mechanical property. That is, the reinforcing effect of GO or FGO on the mechanical properties is similar to that observed for GNP or 4GNP; the improvement is ranked as $G_{12}$ > $G_{23}$ > $E_{22}$ > $E_{11}$ (see Figure 8 and Figure 10). In general, the inclusion of any of the graphene nanoplatelet types results in a complementary contribution to the rigidity of the unidirectional CF composite laminates, which promotes their resistance to bending. This is particularly true for composite elastic properties, which are highly dependent on the matrix/reinforcement interfacial load transfer ($G_{12}$, $G_{23}$, $E_{22}$). 

**Figure 11:**
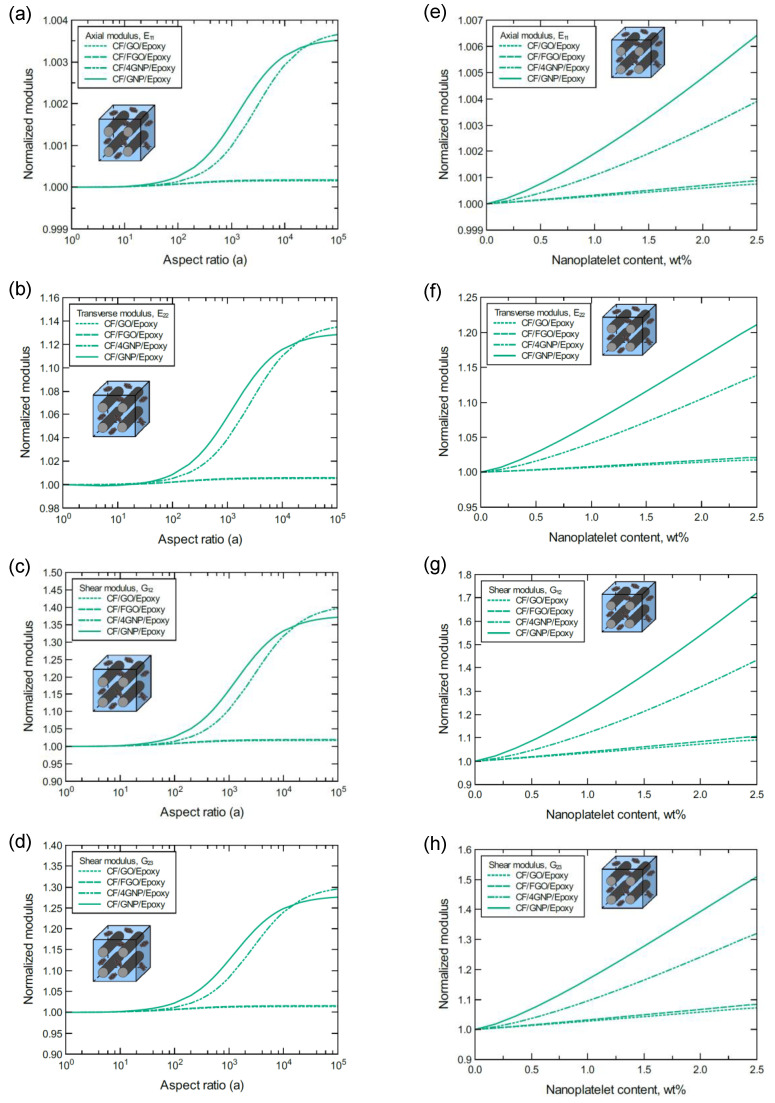
The reinforcing effect of nanoplatelets on the predicted mechanical properties for the hybrid composites (**a**–**d**) at 1.0 wt% nanoplatelet content for various aspect ratio values, (**e**–h) at 10^3^ aspect ratio for various nanoplatelet contents. The volume fraction of CF is 56%.

Figure 11 shows four set of plots to separately compare the improvement in each mechanical property based on the hybrid composite type. Each set of plots provides the response of a specific mechanical property obtained at 1.0 wt% nanoplatelet content for various aspect ratios and at a 10^3^ aspect ratio for various nanoplatelet contents. To emphasize the reinforcing effect, each predicted mechanical property was normalized by its initial magnitude. In general, the reinforcing effect of GNP is the greatest among all the nanoplatelets. While the reinforcing effect of the 4GNP is slightly lower than that of GNP, FGO followed by GO have the lowest reinforcing effect. This is true for the aspect ratio range 10^2^–10^4^. For aspect ratios less than 10^2^, which are most common in nanocomposites, all the nanoplatelet types involve a comparable reinforcing function. Interestingly, at very high aspect ratios (>10^4^), the predicted reinforcing effect of 4GNP slightly surpasses that of GNP.

Figures S1–S4 show the design map plots of unidirectional CF/nanoplatelet/epoxy hybrid composites, which can be used to optimize the axial and transverse moduli by controlling the CF volume fraction and the nanoplatelet content. The design map graphs were also plotted for different nanoplatelet aspect ratios, which represents an additional factor to optimize the mechanical response. Generally, the plots reveal that both CF and the nanoplatelets have a tremendous impact on the elastic response of the hybrid composite. More specifically, CF content has a direct impact on the axial modulus of the hybrid composite, which significantly increases with increases in the CF vol%. A limited contribution to the improvement in the axial modulus can be attributed to the nanoplatelet content and its aspect ratio. The improvement in the transverse modulus of the hybrid composite is largely dominated by the nanoplatelet content and its aspect ratio. Different levels of improvement in the transverse modulus can be observed depending on the nanoplatelet type, content, and aspect ratio. 

### 3.2. Laminated Composite Structures 

The mechanical response prediction of three different stacking orders of laminated CF/nanoplatelet/epoxy hybrid composites is explored herein. The stacking order of each laminate is:

- Symmetric balanced cross-ply laminated composite plate (eight layers)
  [0/90/0/90/90/0/90/0] ≡ [0/90/0/90]_s_ ≡ CP-8;
- Symmetric balanced angle-ply laminated composite plate (eight layers)
  [45/0/−45/90/90/−45/0/45] ≡ [45/0/−45/90]_s_ ≡ AP-8;
- Symmetric balanced angle-ply laminated composite plate (six layers)
  [60/−60/0/0/−60/60] ≡ [60/−60/0]_s_ ≡ AP-6.

The hybrid composite laminate structures were modeled using MAC/GMC. The modeling approach involves two steps, as shown in Figure 2d,e. In the modeling scripts, the predicted mechanical properties from each of the unidirectional CF/nanoplatelets/epoxy hybrid composite types were employed as the baseline properties for the corresponding hybrid composite laminate structure. That is, for a specific structure of the laminated hybrid composite, each lamina represents a unidirectional hybrid composite in which the angle of orientation of the CF reinforcement was assigned based on the above-given set of angles. As a result, the given stacking sequence for the composite laminates in the hybrid composite structure was maintained. It was assumed that each lamina in the laminated composite plate had a thickness of 0.25 mm, and the CF volume fraction was 56%.

Figures S5–S7 show the predicted mechanical properties of the three CF/GNP/epoxy hybrid laminates. Generally, there is improvement in the stiffnesses with increases in the GNP content and aspect ratio. Likewise, the predicted mechanical properties of the laminated composite plates using the CF/4GNP/epoxy hybrid composite are shown in Figures S8–S10. Due to the nanoplatelet agglomeration in the 4GNP system, lower levels of improvement in mechanical properties can be observed relative to the CF/GNP/epoxy laminated composite. Both CF/GO/epoxy-laminated (Figures S11–S13) and CF/FGO/epoxy-laminated (Figures S14–S16) composite plates exhibit nearly identical mechanical responses. The improvement in the predicted mechanical properties of the laminated composite plates using functionalized GNP is limited in comparison to the laminated composite plates using perfectly dispersed pristine GNP. 

Figures S17–S21 show the predicted mechanical properties of the laminated hybrid composite structures with an aspect ratio of 100 for a range of nanoplatelet contents. Each plot compares the predicted elastic mechanical properties from the three laminates using the different nanoplatelet types. In general, the plots demonstrate that the effectiveness of the reinforcement is greater for the angle-ply laminates than for the cross-ply laminates as regards the extensional stiffness (A_11_), the bending stiffness (D_11_), the in-plane elastic modulus (E_xx_ = E_yy_), and the in-plane Poisson’s ratio (ν_xy_). The reinforcement effect is greater for the cross-ply laminates in terms of the shear modulus (G_xy_). 

**Figure 12:**
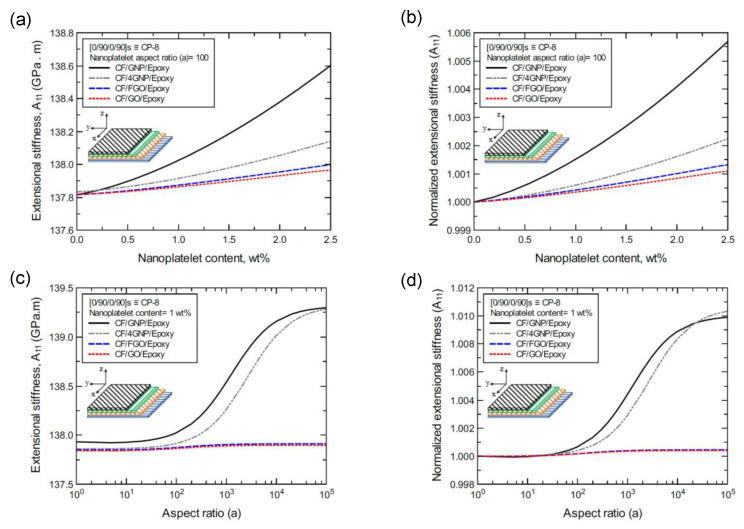
Comparison of the predicted extensional stiffness (A_11_) for a [0/90/0/90]s (CP-8)-laminated composite plate based on the nanoplatelet type, content, and aspect ratio; (**a**) predicted A_11_ for various nanoplatelet contents at 100 aspect ratio, and the normalized response is shown in (**b**); (**c**) predicted A_11_ for various aspect ratio values at 1.0 wt% nanoplatelet content, and the normalized response is shown in (**d**). The volume fraction of CF is 56%.

**Figure 13:**
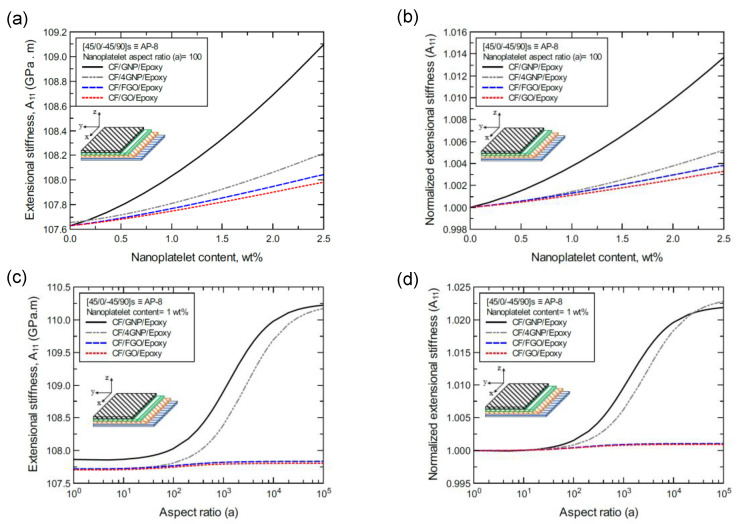
Comparison of the predicted extensional stiffness (A_11_) for a [45/0/-45/90]s (AP-8)-laminated composite plate based on the nanoplatelet type, content, and aspect ratio; (**a**) predicted A_11_ for various nanoplatelet contents at 100 aspect ratio, and the normalized response is shown in (**b**); (**c**) predicted A_11_ for various aspect ratio values at 1.0 wt% nanoplatelet content, and the normalized response is shown in (**d**). The volume fraction of CF is 56%.

**Figure 14:**
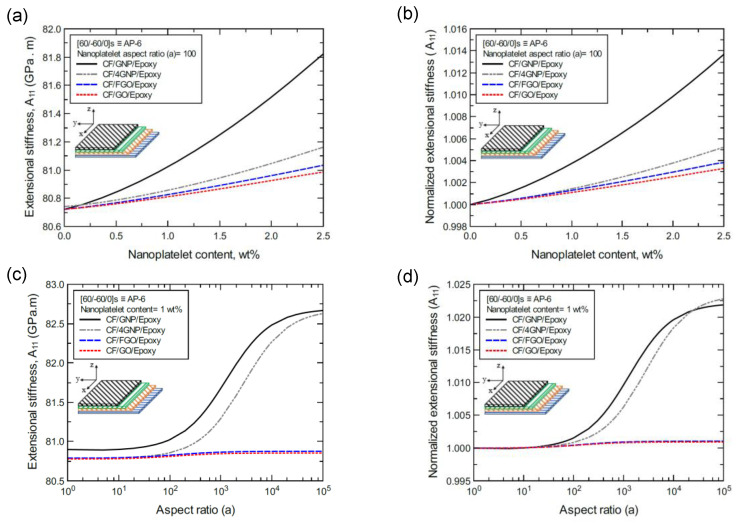
Comparison of the predicted extensional stiffness (A_11_) for a [60/-60/0]s (AP-6)-laminated composite plate based on the nanoplatelet type, content, and aspect ratio; (**a**) predicted A_11_ for various nanoplatelet contents at 100 aspect ratio, and the normalized response is shown in (**b**); (**c**) predicted A_11_ for various aspect ratio values at 1.0 wt% nanoplatelet content, and the normalized response is shown in (**d**). The volume fraction of CF is 56%.

**Figure 15:**
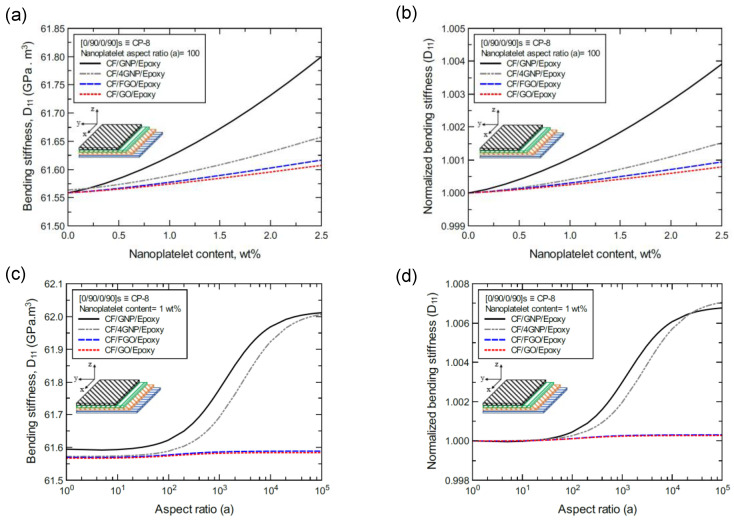
Comparison of the predicted bending stiffness (D_11_) for a [0/90/0/90]s (CP-8)-laminated composite plate based on the nanoplatelet type, content, and aspect ratio; (**a**) predicted D_11_ for various nanoplatelet contents at 100 aspect ratio, and the normalized response is shown in (**b**); (**c**) predicted D_11_ for various aspect ratio values at 1.0 wt% nanoplatelet content, and the normalized response is shown in (**d**). The volume fraction of CF is 56%.

**Figure 16:**
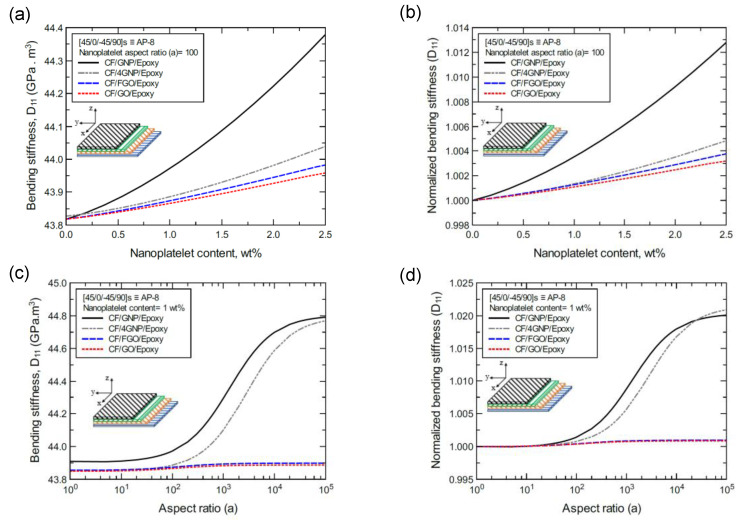
Comparison of the predicted bending stiffness (D_11_) for a [45/0/-45/90]s (AP-8)-laminated composite plate based on the nanoplatelet type, content, and aspect ratio; (**a**) predicted D_11_ for various nanoplatelet contents at 100 aspect ratio, and the normalized response is shown in (**b**); (**c**) predicted D_11_ for various aspect ratio values at 1.0 wt% nanoplatelet content, and the normalized response is shown in (**d**). The volume fraction of CF is 56%.

**Figure 17:**
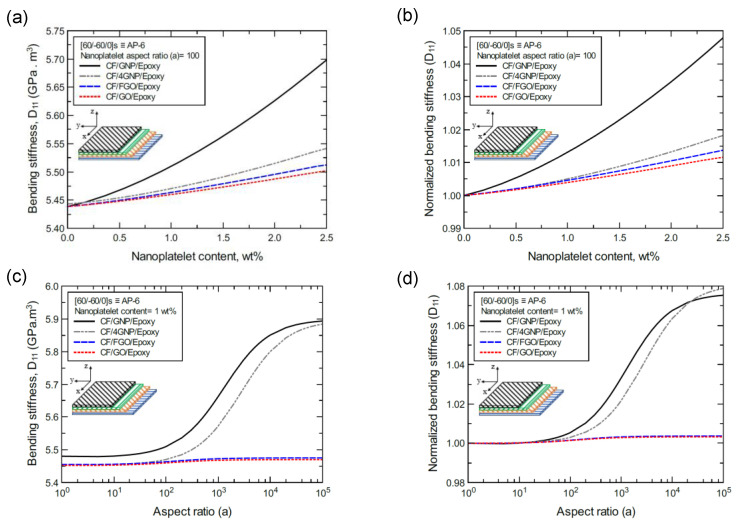
Comparison of the predicted bending stiffness (D_11_) for a [60/-60/0]s (AP-6)-laminated composite plate based on the nanoplatelet type, content, and aspect ratio; (**a**) predicted D_11_ for various nanoplatelet contents at 100 aspect ratio, and the normalized response is shown in (**b**); (**c**) predicted D_11_ for various aspect ratio values at 1.0 wt% nanoplatelet content, and the normalized response is shown in (**d**). The volume fraction of CF is 56%.

**Figure 18:**
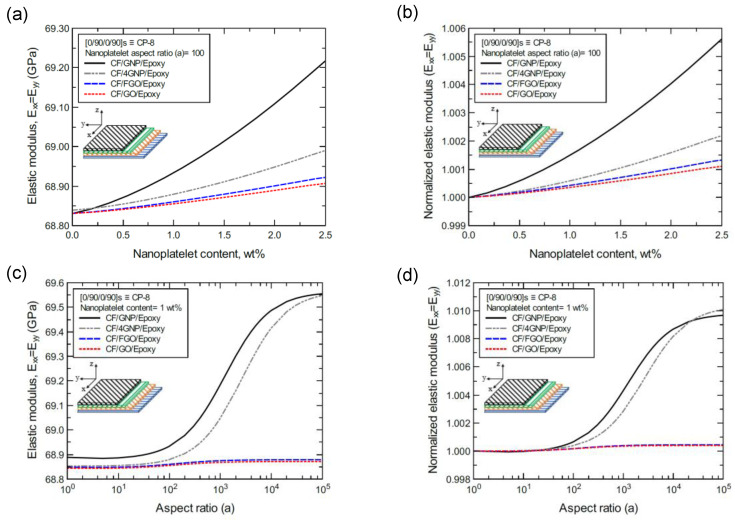
Comparison of the predicted in-plane elastic modulus (E_xx_ = E_yy_) for a [0/90/0/90]s (CP-8)-laminated composite plate based on the nanoplatelet type, content, and aspect ratio; (**a**) predicted E_xx_ for various nanoplatelet contents at 100 aspect ratio, and the normalized response is shown in (**b**); (**c**) predicted E_xx_ for various aspect ratio values at 1.0 wt% nanoplatelet content, and the normalized response is shown in (**d**). The volume fraction of CF is 56%.

**Figure 19:**
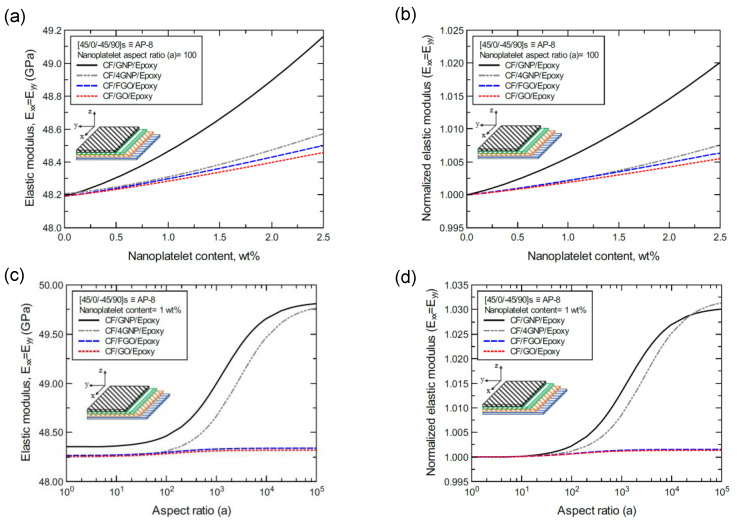
Comparison of the predicted in-plane elastic modulus (E_xx_ = E_yy_) for a [45/0/−45/90]s (AP-8)-laminated composite plate based on the nanoplatelet type, content, and aspect ratio; (**a**) predicted E_xx_ for various nanoplatelet contents at 100 aspect ratio, and the normalized response is shown in (**b**); (**c**) predicted E_xx_ for various aspect ratio values at 1.0 wt% nanoplatelet content, and the normalized response is shown in (**d**). The volume fraction of CF is 56%.

**Figure 20:**
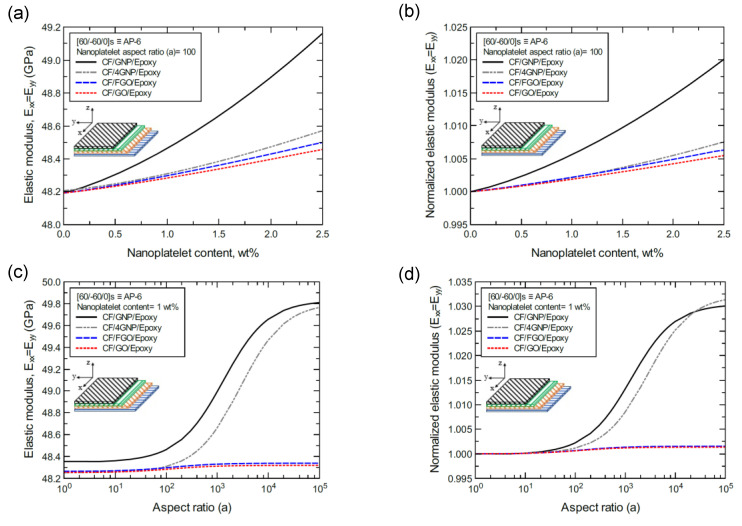
Comparison of the predicted in-plane elastic modulus (E_xx_ = E_yy_) for a [60/−60/0]s (AP-6)-laminated composite plate based on the nanoplatelet type, content, and aspect ratio; (**a**) predicted E_xx_ for various nanoplatelet contents at 100 aspect ratio, and the normalized response is shown in (**b**); (**c**) predicted E_xx_ for various aspect ratio values at 1.0 wt% nanoplatelet content, and the normalized response is shown in (**d**). The volume fraction of CF is 56%.

**Figure 21:**
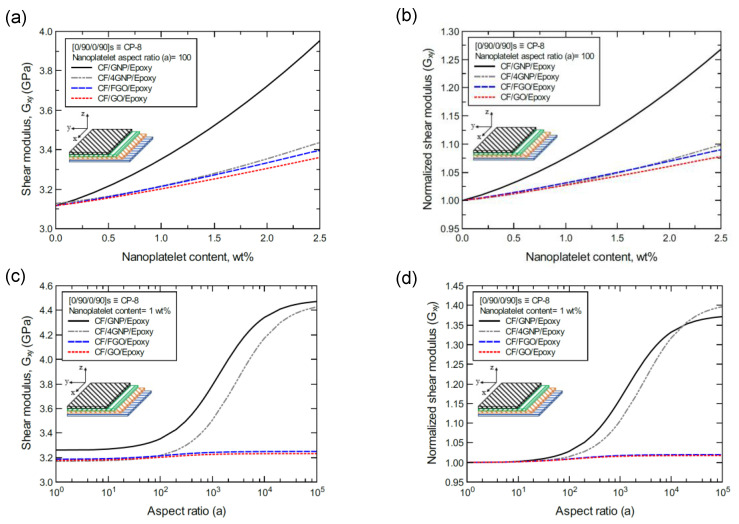
Comparison of the predicted in-plane shear modulus (G_xy_) for a [0/90/0/90]s (CP-8)-laminated composite plate based on the nanoplatelet type, content, and aspect ratio; (**a**) predicted G_xy_ for various nanoplatelet contents at 100 aspect ratio, and the normalized response is shown in (**b**); (**c**) predicted G_xy_ for various aspect ratio values at 1.0 wt% nanoplatelet content, and the normalized response is shown in (**d**). The volume fraction of CF is 56%.

**Figure 22:**
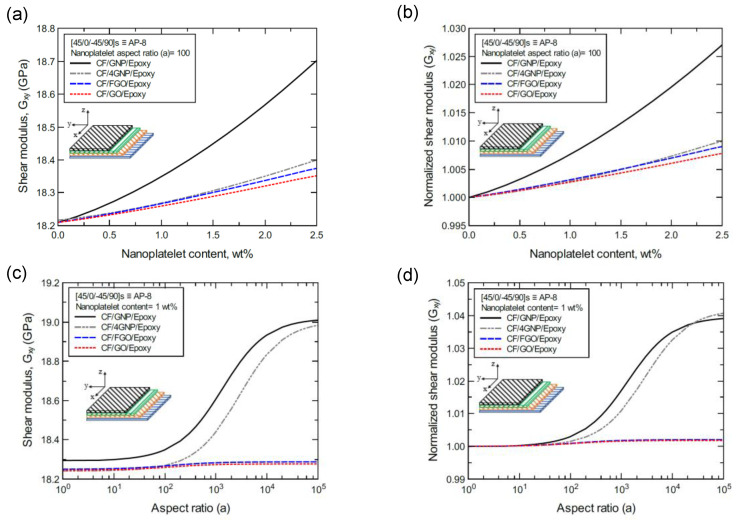
Comparison of the predicted in-plane shear modulus (G_xy_) for a [45/0/-45/90]s (AP-8)-laminated composite plate based on the nanoplatelet type, content, and aspect ratio; (**a**) predicted G_xy_ for various nanoplatelet contents at 100 aspect ratio, and the normalized response is shown in (**b**); (**c**) predicted G_xy_ for various aspect ratio values at 1.0 wt% nanoplatelet content, and the normalized response is shown in (**d**). The volume fraction of CF is 56%.

**Figure 23:**
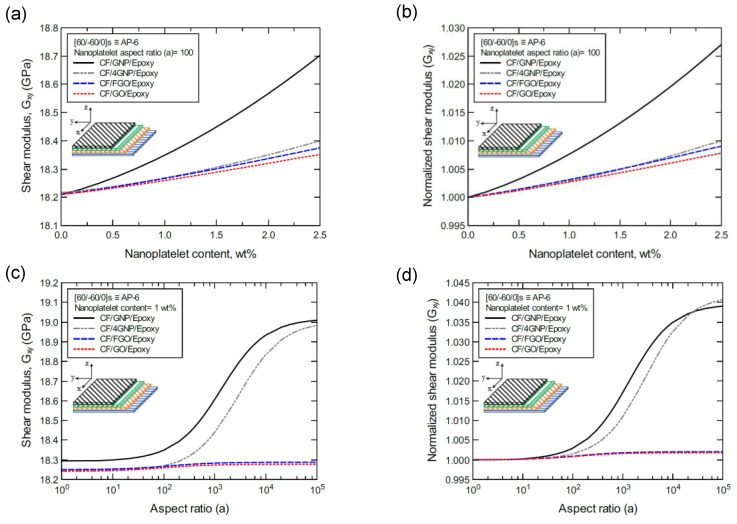
Comparison of the predicted in-plane shear modulus (G_xy_) for a [60/-60/0]s (AP-6)-laminated composite plate based on the nanoplatelet type, content, and aspect ratio; (**a**) predicted G_xy_ for various nanoplatelet contents at 100 aspect ratio, and the normalized response is shown in (**b**); (**c**) predicted G_xy_ for various aspect ratio values at 1.0 wt% nanoplatelet content, and the normalized response is shown in (**d**). The volume fraction of CF is 56%.

**Figure 24:**
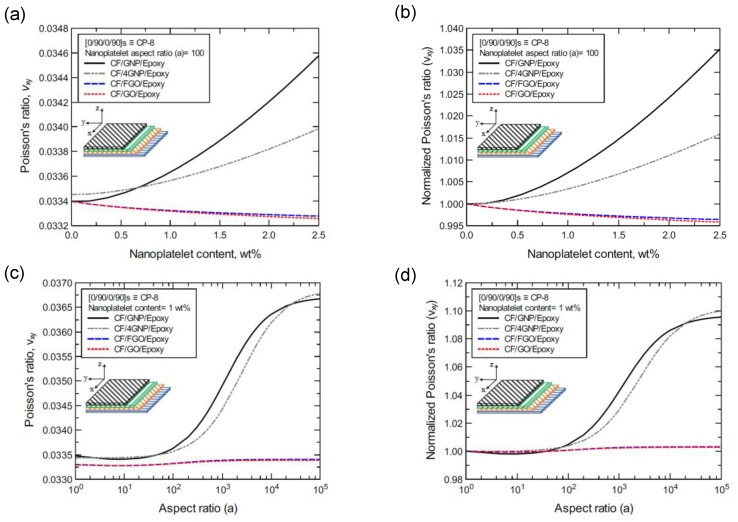
Comparison of the predicted in-plane Poisson’s ratio (ν_xy_) for a [0/90/0/90]s (CP-8)-laminated composite plate based on the nanoplatelet type, content, and aspect ratio; (**a**) predicted ν_xy_ for various nanoplatelet contents at 100 aspect ratio, and the normalized response is shown in (**b**); (**c**) predicted ν_xy_ for various aspect ratio values at 1.0 wt% nanoplatelet content, and the normalized response is shown in (**d**). The volume fraction of CF is 56%.

**Figure 25:**
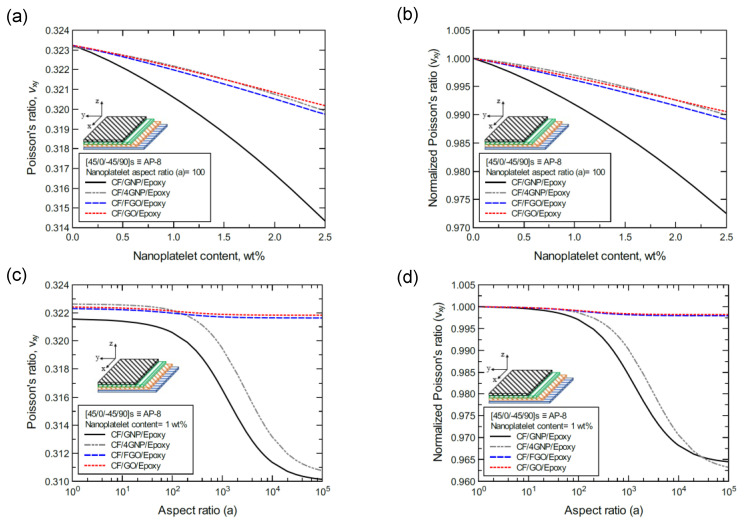
Comparison of the predicted in-plane Poisson’s ratio (ν_xy_) for a [45/0/-45/90]s (AP-8)-laminated composite plate based on the nanoplatelet type, content, and aspect ratio; (**a**) predicted ν_xy_ for various nanoplatelet contents at 100 aspect ratio, and the normalized response is shown in (**b**); (**c**) predicted ν_xy_ for various aspect ratio values at 1.0 wt% nanoplatelet content, and the normalized response is shown in (**d**). The volume fraction of CF is 56%.

**Figure 26:**
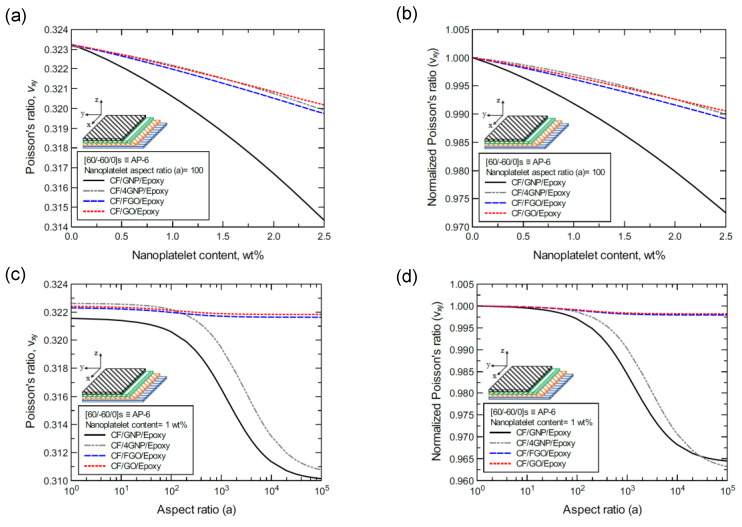
Comparison of the predicted in-plane Poisson’s ratio (ν_xy_) for a [60/-60/0]s (AP-6)-laminated composite plate based on the nanoplatelet type, content, and aspect ratio; (**a**) predicted ν_xy_ for various nanoplatelet contents at 100 aspect ratio, and the normalized response is shown in **(b**); (**c**) predicted ν_xy_ for various aspect ratio values at 1.0 wt% nanoplatelet content, and the normalized response is shown in (**d**). The volume fraction of CF is 56%.

To better illustrate the reinforcing effect of each of the four nanoplatelet types on the mechanical response of the laminated hybrid composite plates, Figure 12, Figure 13, Figure 14, Figure 15, Figure 16, Figure 17, Figure 18, Figure 19, Figure 20, Figure 21, Figure 22, Figure 23, Figure 24, Figure 25 and Figure 26 show comparison plots of each of the predicted mechanical properties based on the nanoplatelet type, content, and aspect ratio. This comparison was performed on the three proposed structures of the laminated hybrid composite plates with a CF volume fraction of 56%. It can be generalized that the reinforcing effect of the nanoplatelets follows the order GNP > 4GNP > FGO ≥ GO.

Figure 12a shows the predicted extensional stiffness (A_11_) values of the CP-8-laminated hybrid composite plate, along with the normalized values shown in Figure 12b. The comparison between A_11_ values is based on the nanoplatelet type and content with an aspect ratio of 100. Figure 12c shows the A_11_ values for the same laminated hybrid composite plate, along with the normalized values shown in Figure 12d, which are predicted for various nanoplatelet aspect ratios for a nanoplatelet content of 1.0 wt%. Clearly, the trend in the nanoplatelet reinforcing effect based on the predicted A_11_ values using each of the four nanoplatelet types follows the order of GNP > 4GNP > FGO ≥ GO. However, this rule does not hold true at conditions with a low nanoplatelet content (<1 wt%) and aspect ratio (< 100), for which there are nearly identical reinforcing effects. Another exception to this rule occurs in the rare instance when the aspect ratio is greater than 10^4^, at which point the reinforcing effect of 4GNP surpasses that of GNP (Figure 12d). Similar trends can be observed for the predicted A_11_ values of the AP-8 and AP-6 laminated hybrid composite plates shown in Figure 13 and Figure 14, respectively. 

In the same manner, Figure 15a shows the predicted bending stiffness (D_11_) values of the CP-8-laminated hybrid composite plate, along with the normalized values, which are shown in Figure 15b. The comparison between D_11_ values is based on the nanoplatelet type and content with a constant aspect ratio of 100. Figure 15c shows the D_11_ values for the same laminated hybrid composite plate, and the normalized values are shown in Figure 15d, which are predicted for various nanoplatelet aspect ratio values and a constant nanoplatelet content of 1.0 wt%. Considering the exceptions mentioned above, the trend in the nanoplatelet reinforcing effect based on the predicted D_11_ values using each of the four nanoplatelet types also follows the order of GNP > 4GNP > FGO ≥ GO. Similar trends can be observed for the predicted D_11_ values of the AP-8- and AP-6-laminated hybrid composites shown in Figure 16 and Figure 17, respectively. 

Figure 18a shows the predicted elastic modulus (E_xx_ = E_yy_) values of the CP-8-laminated hybrid composite plate, along with the normalized values shown in Figure 18b. The comparison between E_xx_ values is based on the nanoplatelet type and content, with a constant aspect ratio of 100. Figure 18c shows the E_xx_ values for the same laminated hybrid composite plate, along with the normalized values shown in Figure 18d, which are predicted for various nanoplatelet aspect ratio values and a constant nanoplatelet content of 1.0 wt%. The trend in the nanoplatelet reinforcing effect based on the predicted E_xx_ values using each of the four nanoplatelet types follows the order of GNP > 4GNP > FGO ≥ GO. Similar trends can be observed for the predicted E_xx_ values of the AP-8- and AP-6-laminated hybrid composites, as shown in Figure 19 and Figure 20, respectively. More specifically, the reinforcing effects of 4GNP, FGO, and GO based on the predicted E_xx_ values are more likely to be close or identical at nanoplatelet aspect ratio values less than 100.

Figure 21a shows the predicted shear modulus (G_xy_) values of the CP-8-laminated hybrid composite, along with the normalized values shown in Figure 21b. The comparison between G_xy_ values is based on the nanoplatelet type (GNP, 4GNP, FGO, or GO) with various nanoplatelet contents and an aspect ratio of 100. Figure 21c shows the G_xy_ values for the same laminated hybrid composite plate, along with the normalized values shown in Figure 21d, which are predicted for various nanoplatelet aspect ratio values and a constant nanoplatelet content of 1.0 wt%. The trend in the nanoplatelet reinforcing effect based on the predicted G_xy_ values using each of the four nanoplatelet types follows the order of GNP > 4GNP > FGO ≥ GO. Similar trends can be observed for the predicted G_xy_ values of the AP-8- and AP-6-laminated hybrid composites shown in Figure 22 and Figure 23, respectively. More specifically, the reinforcing effects of the 4GNP, FGO, and GO systems based on the predicted G_xy_ values are more likely to be close or identical at nanoplatelet aspect ratio values less than 100.

Finally, Figure 24a shows the predicted Poisson’s ratio (ν_xy_) values of the CP-8-laminated hybrid composite, along with the normalized values shown in Figure 24b. The comparison between ν_xy_ values is based on the nanoplatelet type content with an aspect ratio of 100. Figure 24c shows the ν_xy_ values for the same laminated hybrid composite plate, along with the normalized values shown in Figure 24d, which are predicted for various nanoplatelet aspect ratios with a nanoplatelet content of 1.0 wt%. As the inclusion of GNP and 4GNP produces an increase in the predicted ν_xy_, identical ν_xy_ values (with slight reductions for aspect ratio values < 100, and slight increases for aspect ratio values > 100) can be observed for the inclusion of FGO and GO nanoplatelets. Similarly, Figure 25 and Figure 26 show the ν_xy_ values predicted for the AP-8 and AP-6 composites, respectively. The plots show a general decrease in the predicted ν_xy_ as the nanoplatelet content and aspect ratio are increased. For aspect ratio values < 100, nearly identical responses can be observed for the predicted ν_xy_ with 4GNP, FGO, and GO. However, the decrease in the predicted ν_xy_ with 4GNP is close to that predicted with GNP, with aspect ratio values > 100. The ν_xy_ values predicted with GNP registered the highest drop among the nanoplatelets, except at very large aspect ratio values, where the drop in ν_xy_ with 4GNP surpassed that with GNP. 

## 4. Summary and Conclusions

A parametric computational study was performed to assess the mechanical performance of aerospace epoxy composites reinforced with carbon fiber and functionalized graphene nanoplatelets. Four types of graphene nanoplatelets were considered (pristine, agglomerated, oxidized, and functionalized), and a wide range of nanoplatelet loadings and aspect ratios were also used as adjustable parameters. Micromechanical modeling methods were used to predict the effects of these parameters on the overall laminate properties for unidirectional, cross-ply, and angle-ply layups. 

For the unidirectional CF/nanoplatelet/epoxy hybrid composites, the predicted mechanical properties indicate that the axial mechanical response is dominated by the CF. However, there is an improvement in the transverse mechanical response owing to the nanoplatelet reinforcing effect. For the cross-ply and angle-ply hybrid laminates, the reinforcing effect of the nanoplatelets is rather insignificant in terms of the in-plane mechanical response. This is because the in-plane mechanical properties are highly governed by the angle or orientation of the CF within each lamina. 

Based upon the engineering application or the mechanical function of the structural component, this comparative study can provide insight for designing and fabricating hybrid laminated composite structures using graphene nanoplatelets. The results of these models can be utilized to optimize the mechanical behavior of the laminated hybrid composite by controlling the parameters that affect the mechanical response. These controlling parameters can be adjusted according to the component function within the structure. The CF angle/direction in each lamina and the number of laminas and their stacking sequence are fundamental to determining the mechanical response of the constructed laminated composite. The nanoplatelet content and its aspect ratio are additional important factors that can be considered in fabricating laminated hybrid composite plates. 

## Figures and Tables

**Table 1:** The predicted elastic properties of graphene nanoplatelet/epoxy nanocomposite MD models (effective mechanical properties of the locale interphase region).

Mechanical Properties	GNP/Epoxy [42]	4GNP/Epoxy [1]	GO/EPOXY [42]	FGO/Epoxy [42]
$\mathrm{In}-\mathrm{plane}\;\mathrm{elastic}\;\mathrm{modulus}\;(E_{ip}$), GPa	127.5 ± 1.6	420.5 ± 2.5	13.7 ± 2.3	14.1 ± 2.2
$\mathrm{Out}-\mathrm{of}-\mathrm{plane}\;\mathrm{elastic}\;\mathrm{modulus}\;(E_{op}$), GPa	5.1 ± 0.5	5.3 ± 0.6	3.8 ± 0.9	4.2 ± 0.5
$\mathrm{In}-\mathrm{plane}\;\mathrm{shear}\;\mathrm{modulus}\;(G_{ip}$), GPa	30.1 ± 0.9	102.0 ± 1.0	7.7 ± 1.1	8.3 ± 0.8
$\mathrm{Out}-\mathrm{of}-\mathrm{plane}\;\mathrm{shear}\;\mathrm{modulus}\;(G_{op}$), GPa	0.073 ± 0.021	0.019 ± 0.007	1.201 ± 0.214	1.498 ± 0.239
$\mathrm{In}-\mathrm{plane}\;\mathrm{Poisson}’s\;\mathrm{ratio}\;(\nu_{ip}$)	0.964 ± 0.003	0.993 ± 0.001	0.080 ± 0.021	0.071 ± 0.043
$\mathrm{Out}-\mathrm{of}-\mathrm{plane}\;\mathrm{Poisson}’s\;\mathrm{ratio}\;(\nu_{op}$)	0.020 ± 0.007	0.002 ± 0.001	0.321 ± 0.067	0.267 ± 0.032

## Data Availability

The data presented in this study are available on request from the corresponding author. The data are not publicly available because they are currently being used for further studies.

## Supplementary Materials

The following are available online at https://www.mdpi.com/article/10.3390/nano11112919/s1, Figures S1–S21. Figures S1-S4 show the design maps of the unidirectional hybrid composites in which the axial and transverse moduli were predicted at different CF vol% and considering the effect of the content and aspect ratio for the four nanoplatelet types. Figures S5-S16 show predicted mechanical properties of the three types of the laminated hybrid composite structures considering the effect of the content and aspect ratio for the four nanoplatelet types. Figures S17-S21 show a comparison for the reinforcing effect of the nanoplatelets content on the predicted mechanical properties for the three types of the laminFFated hybrid composite structures.
