# Supplementary material for: Computational Modeling of Hybrid Carbon Fiber/Epoxy Composites Reinforced with Functionalized and Non-Functionalized Graphene Nanoplatelets
Source: Nanomaterials (Basel). 2021 Oct 31;11(11):2919. doi: 10.3390/nano11112919 (PMC8622381; doi:10.3390/nano11112919)
Supplement: Supplementary file 1 [file nanomaterials-11-02919-s001.zip › nanomaterials-1429292-supplementary.pdf]

nanomaterials

Supplementary Materials

Computational Modeling of Hybrid Carbon Fiber/Epoxy Composites Reinforced with Functionalized and Non-Functionalized Graphene Nanoplatelets

Hashim Al Mahmud [1,2], Matthew S. Radue [2], William A. Pisani [3,4] and Gregory M. Odegard [2,*]

[1] Department of Mechanical Engineering, University of Kufa, P.O. Box 21, Kufa 54003, , Iraq; hashimn.almahmood@uokufa.edu.iq or hnalmahm@mtu.edu
[2] Department of Mechanical Engineering-Engineering Mechanics, Michigan Technological University, Houghton, MI 49931, USA; msradue@mtu.edu
[3] Oak Ridge Institute for Science and Education, Oak Ridge, TN 37830, USA; william.a.pisani@usace.army.mil
[4] US Army Engineer Research and Development Center, Environmental Laboratory, Vicksburg, MS 39180, USA
* Correspondence: gmodegar@mtu.edu

Unidirectional CF/graphene nanoplatelet/epoxy hybrid composite design maps

Figures S1–S4 show design map plots of unidirectional CF/nanoplatelet/epoxy hybrid composites which can be used to optimize the axial and transverse moduli by controlling the CF volume fraction and the nanoplatelet content. The design map graphs were also plotted for different nanoplatelet aspect ratios, which represents an additional factor to optimize the mechanical response. Generally, the plots reveal that both CF and the nanoplatelets have a tremendous impact on the elastic response of the hybrid composite. More specifically, CF content shows a direct impact on the axial modulus of the hybrid composite which significantly increases with increasing the CF vol%. A limited contribution to the improvement in the axial modulus can be attributed to the nanoplatelet content and its aspect ratio.

The improvement in the transverse modulus of the hybrid composite is largely dominated by the nanoplatelet content and its aspect ratio. Different levels of improvement in the transverse modulus can be observed depending on the nanoplatelet type, content, and aspect ratio. The best levels of improvement in the transverse modulus are predicted for the unidirectional CF/GNP/epoxy hybrid composite (Figure S1), which is attributed to the aforementioned ideal dispersion of the GNP/epoxy nanocomposite. The improvement levels in the transverse modulus of the unidirectional CF/4GNP/epoxy composite (Figure S2) is in the second to the GNP hybrid composite as the 4GNP represents a lower level of nanoplatelet dispersion relative to GNP. The improvement levels in the transverse modulus of unidirectional CF/GO/epoxy and CF/FGO/epoxy are significantly lower than those associated with the GNP and 4GNP hybrid composites (Figures S3 and S4). This is mainly attributed to the weak mechanical performance of GO and FGO nanoplatelets relative to the pristine GNP [1]. In addition, the limited reinforcing effect of GO and FGO resulted in a relatively short range of improvement in the transverse modulus of their hybrid composites as the nanoplatelets content and aspect ratio were increased.

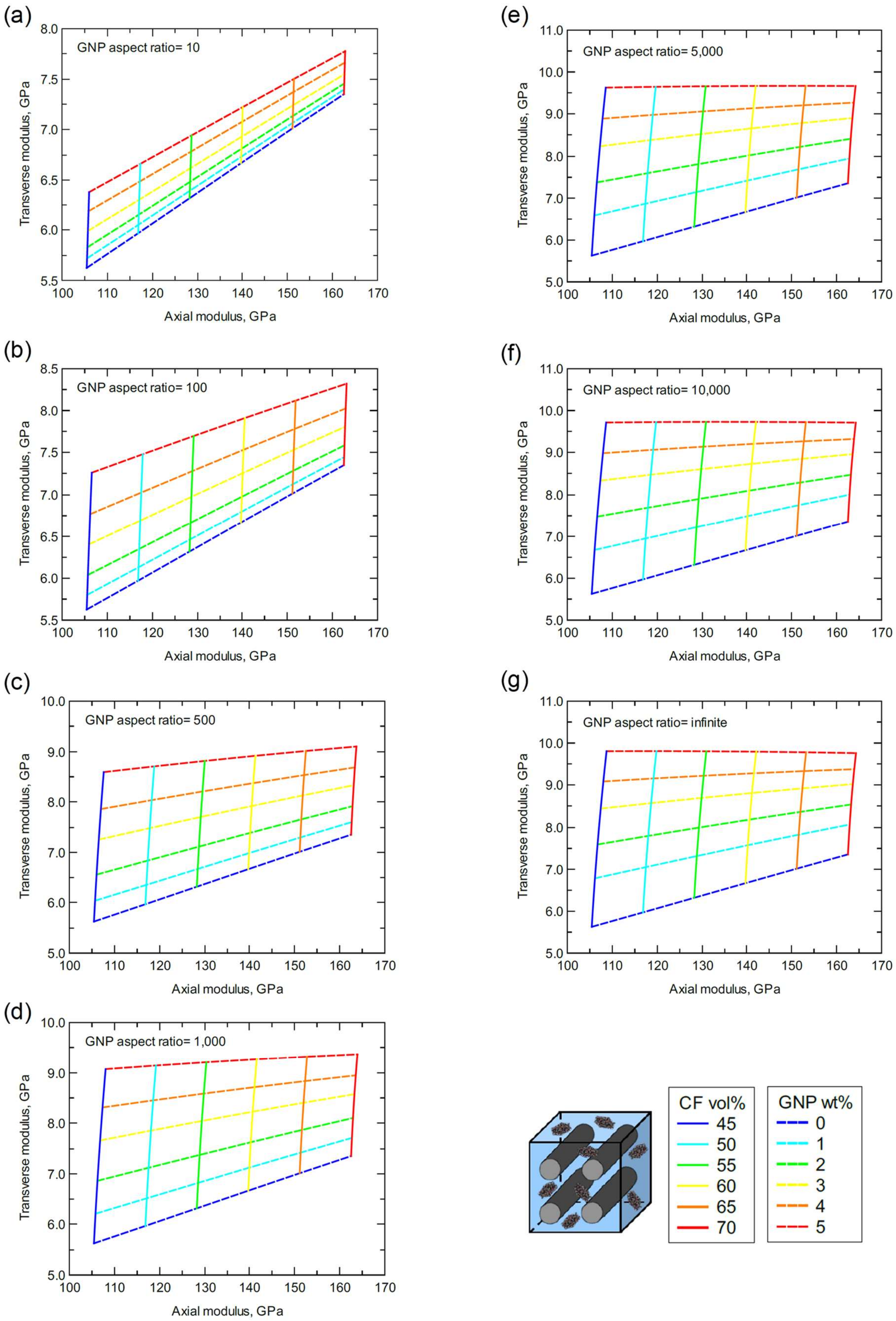

Figure S1. Predicted axial and transverse moduli for unidirectional CF/GNP/epoxy at different CF vol% and GNP wt%; (a) for GNP aspect ratio = 10; (b) for GNP aspect ratio = 100; (c) for GNP aspect ratio = 500; (d) for GNP aspect ratio = 1,000; (e) for GNP aspect ratio = 5,000; (f) for GNP aspect ratio = 10,000; (g) for GNP aspect ratio = infinite.

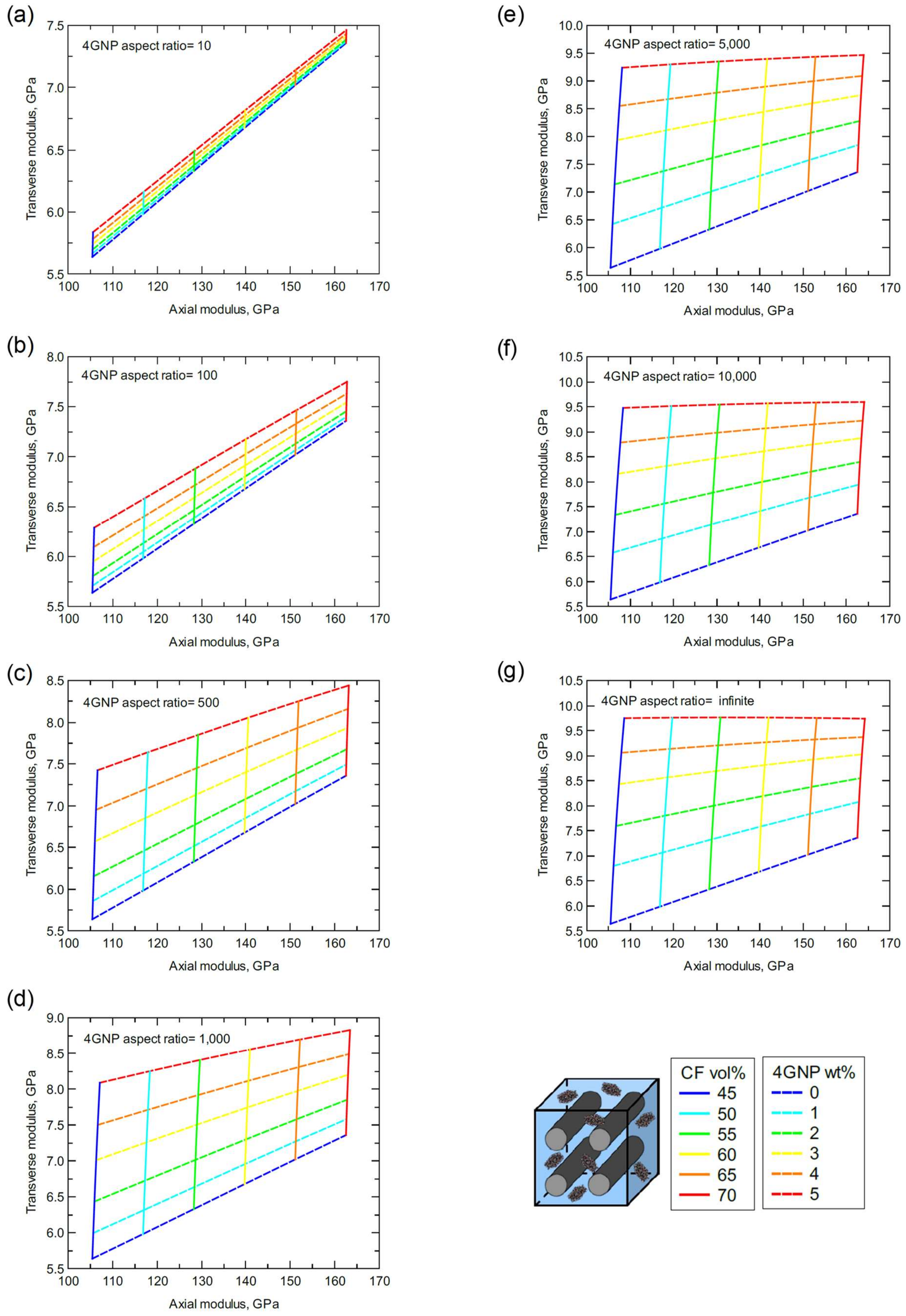

Figure S2. Predicted axial and transverse moduli for unidirectional CF/4GNP/epoxy at different CF vol% and 4GNP wt%; (a) for 4GNP aspect ratio = 10; (b) for 4GNP aspect ratio = 100; (c) for 4GNP aspect ratio = 500; (d) for 4GNP aspect ratio = 1,000; (e) for 4GNP aspect ratio = 5,000; (f) for 4GNP aspect ratio = 10,000; (g) for 4GNP aspect ratio = infinite.

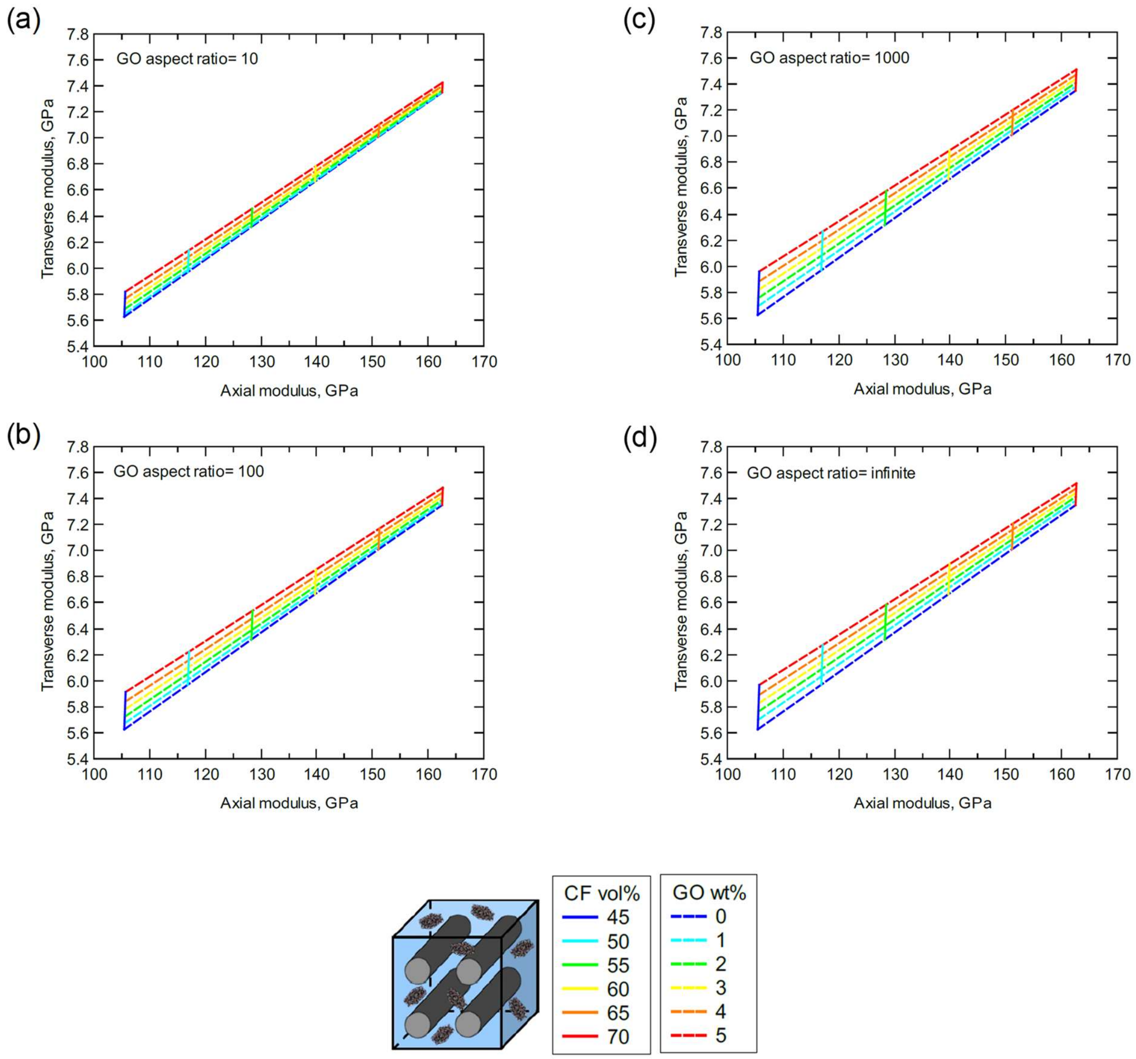

Figure S3. Predicted axial and transverse moduli for unidirectional CF/GO/epoxy at different CF vol% and GO wt%; (a) for GO aspect ratio = 10; (b) for GO aspect ratio = 100; (c) for GO aspect ratio = 1,000; (d) for GO aspect ratio = infinite.

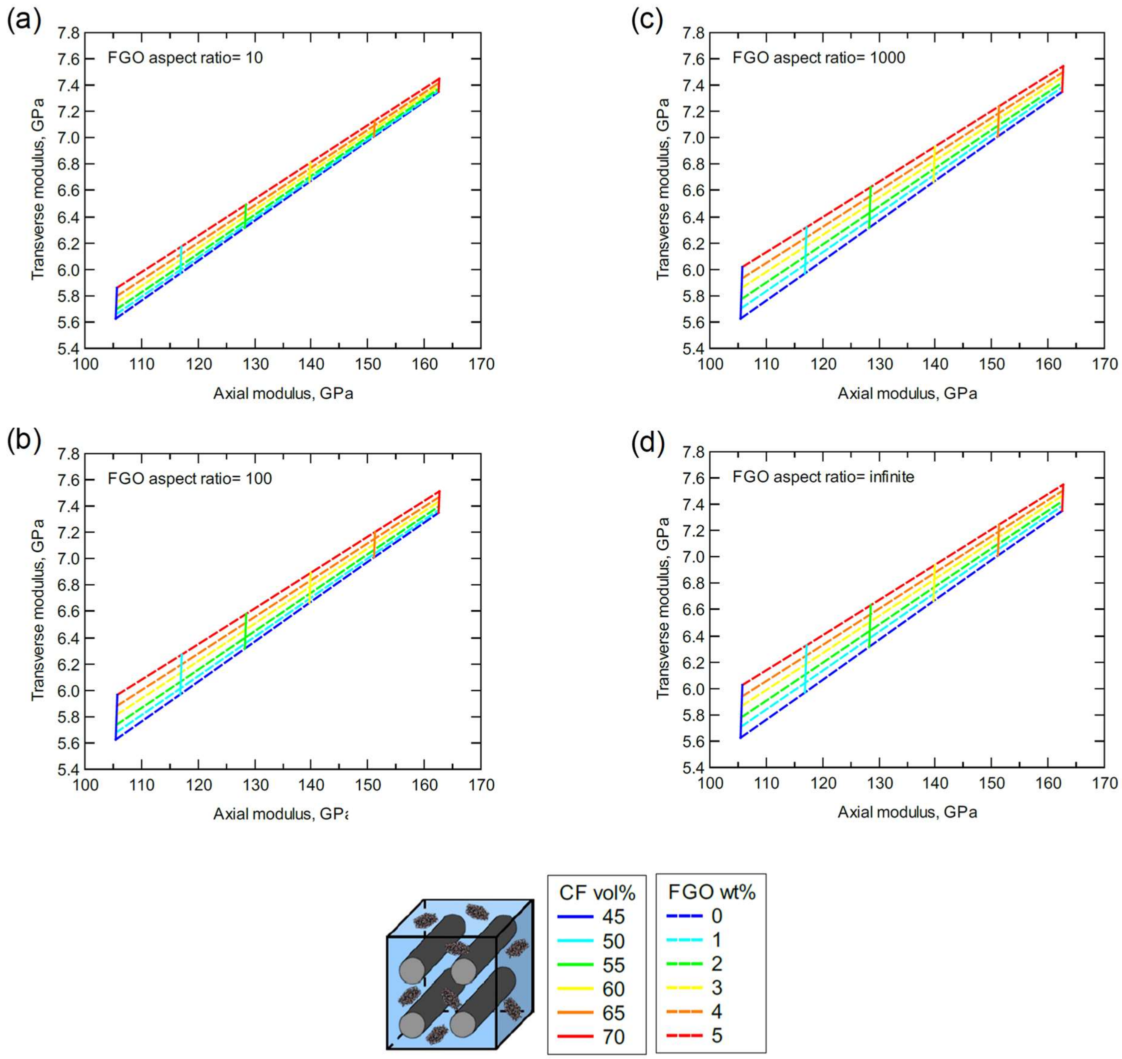

Figure S4. Predicted axial and transverse moduli for unidirectional CF/FGO/epoxy at different CF vol% and FGO wt%; (a) for FGO aspect ratio = 10; (b) for FGO aspect ratio = 100; (c) for FGO aspect ratio = 1,000; (d) for FGO aspect ratio = infinite.

Comparison of properties and aspect ratios for laminated hybrid composites

Figures S5–S7 show the predicted mechanical properties of the three CF/GNP/epoxy hybrid laminates. Generally, there is improvement in the stiffnesses with increasing the GNP content and aspect ratio. Note the coupling stiffness (B_{11}) for symmetric balanced laminated composite plates is always zero. Also, the in-plane elastic moduli E_{xx} and E_{yy} are identical due to the symmetry in laminating order. While the cross-ply composite plate exhibits a slight increase in Poisson's ratio with GNP content and aspect ratio, both angle-ply composite plates show a slight decrease. In all cases, however, the variation in the laminated plate contraction is relatively small. Likewise, the predicted mechanical properties of the laminated composite plates using the CF/4GNP/epoxy hybrid composite are shown in Figures S8–S10. Due to the nanoplatelet agglomeration in the 4GNP system, lower levels of improvement in mechanical properties can be observed relative to the CF/GNP/epoxy laminated composite. Both CF/GO/epoxy (Figures S11–S13) and CF/FGO/epoxy (Figures S14–S16) laminated composite plates exhibit nearly identical mechanical responses. The improvement in the predicted mechanical properties of the laminated composite plates using functionalized GNP is limited in comparison to the laminated composite plates using perfectly dispersed pristine GNP.

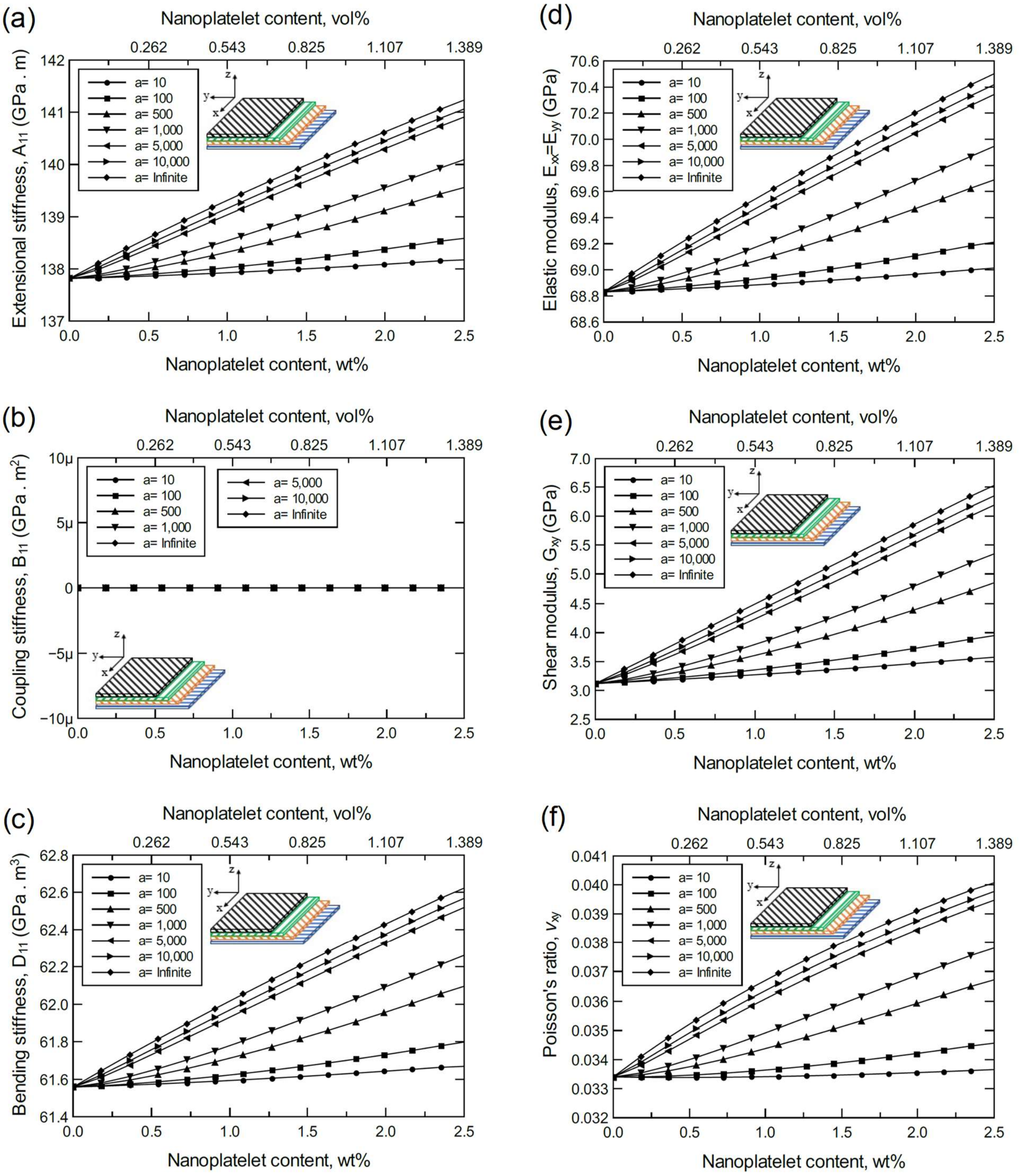

Figure S5. Predicted mechanical properties of the laminated CF/GNP/epoxy, a symmetric balanced cross-ply laminated composite plate $[0/90/0/90]_s$; (**a**) extensional stiffness, A_{11}; (**b**) coupling stiffness, B_{11}; (**c**) bending stiffness, D_{11}; (**d**) elastic modulus, $E_{xx} = E_{yy}$; (**e**) shear modulus, G_{xy}; (**f**) Poisson's ratio, ν_{xy}. The volume fraction of CF is 56%.

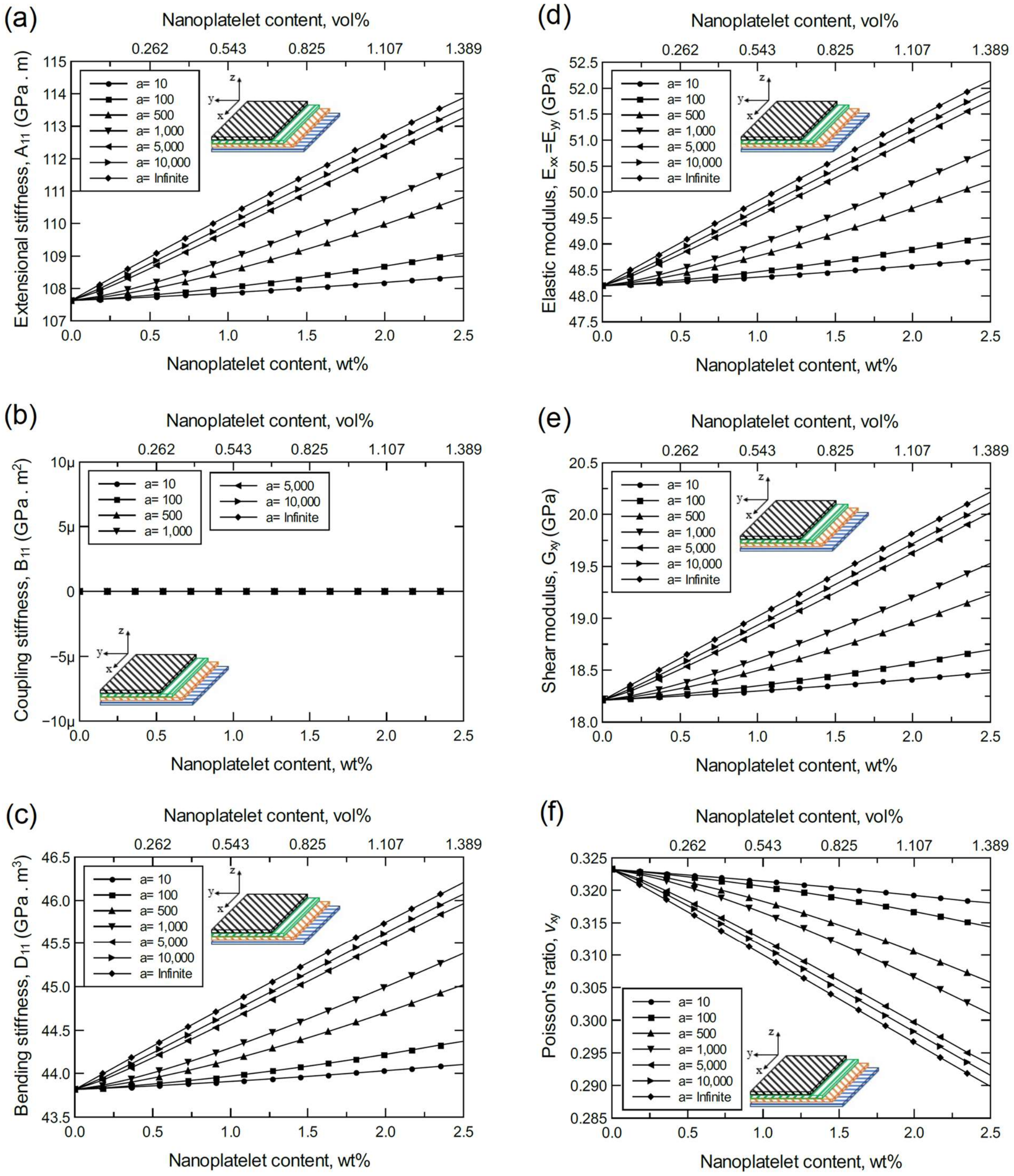

Figure S6. Predicted mechanical properties of the laminated CF/GNP/epoxy, a symmetric balanced angle-ply laminated composite plate $[45/0/-45/90]_s$; (**a**) extensional stiffness, A_{11}; (**b**) coupling stiffness, B_{11}; (**c**) bending stiffness, D_{11}; (**d**) elastic modulus, $E_{xx} = E_{yy}$; (**e**) shear modulus, G_{xy}; (**f**) Poisson's ratio, ν_{xy}. The volume fraction of CF is 56%.

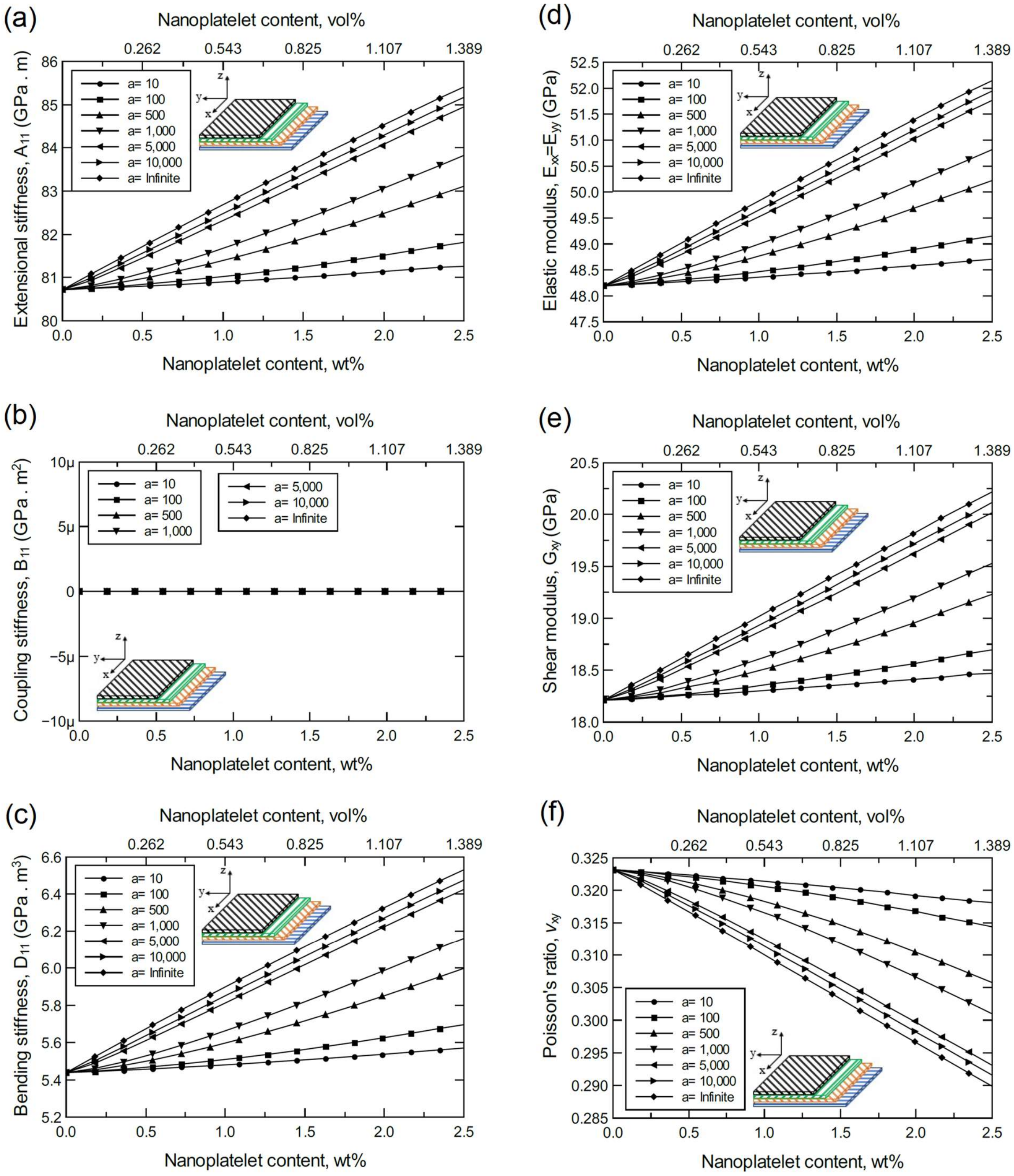

Figure S7. Predicted mechanical properties of the laminated CF/GNP/epoxy, a symmetric balanced angle-ply laminated composite plate $[60/-60/0]_s$; (**a**) extensional stiffness, A_{11}; (**b**) coupling stiffness, B_{11}; (**c**) bending stiffness, D_{11}; (**d**) elastic modulus, $E_{xx} = E_{yy}$; (**e**) shear modulus, G_{xy}; (**f**) Poisson's ratio, ν_{xy}. The volume fraction of CF is 56%.

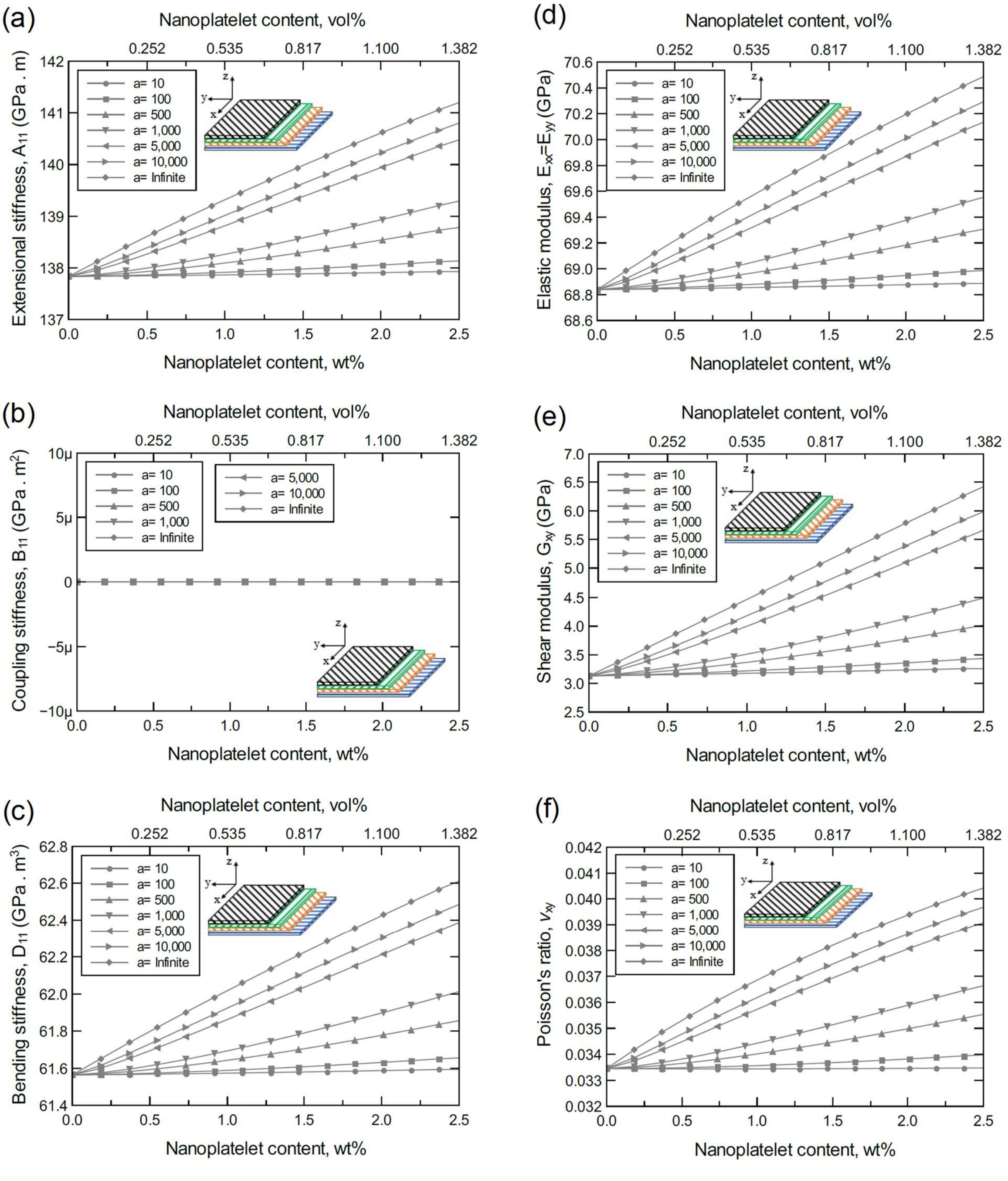

Figure S8. Predicted mechanical properties of the laminated CF/4GNP/epoxy, a symmetric balanced cross-ply laminated composite plate [0/90/0/90]$_s$; (**a**) extensional stiffness, A_{11}; (**b**) coupling stiffness, B_{11}; (**c**) bending stiffness, D_{11}; (**d**) elastic modulus, $E_{xx} = E_{yy}$; (**e**) shear modulus, G_{xy}; (**f**) Poisson's ratio, ν_{xy}. The volume fraction of CF is 56%.

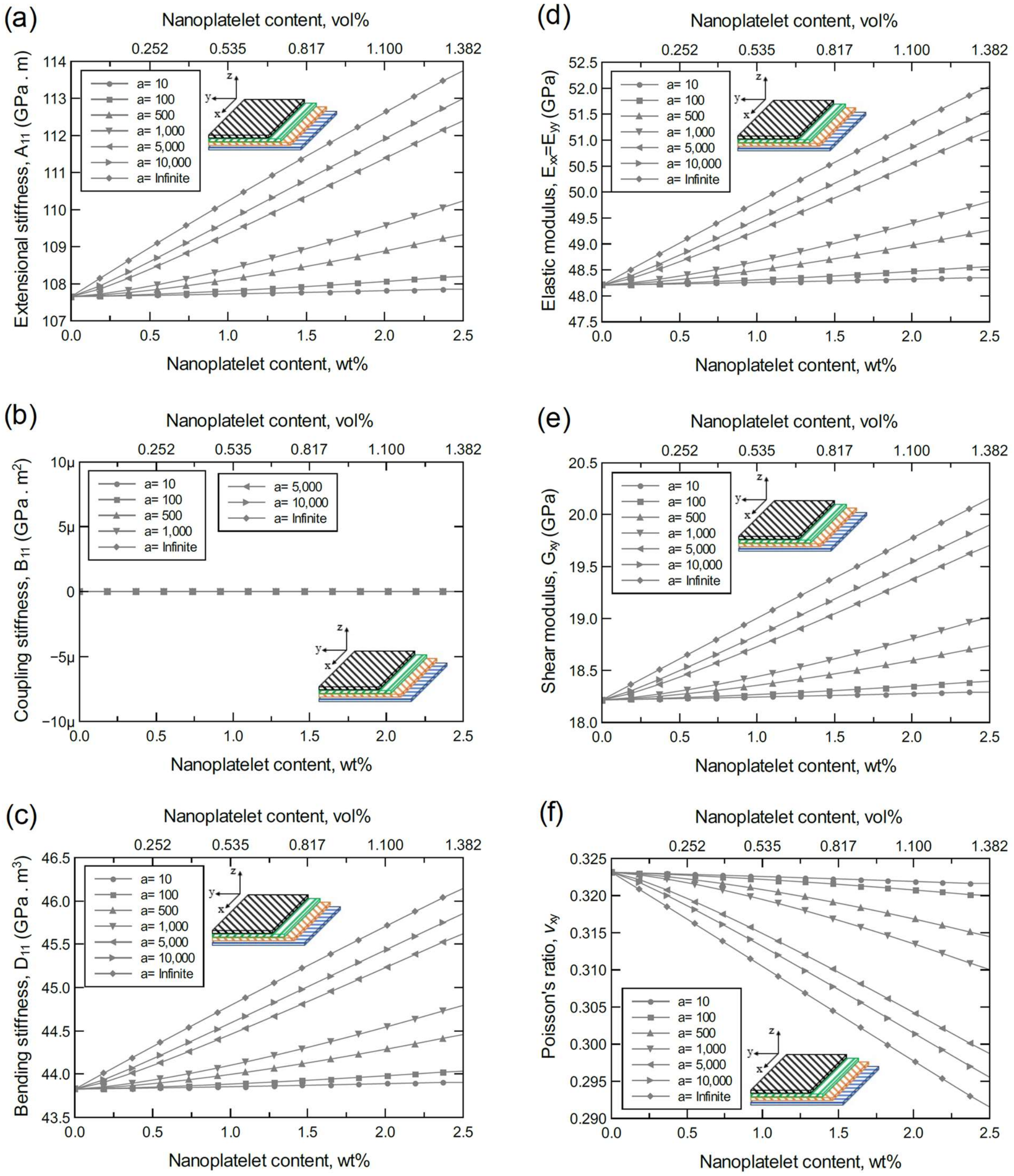

Figure S9. Predicted mechanical properties of the laminated CF/4GNP/epoxy, a symmetric balanced angle-ply laminated composite plate $[45/0/-45/90]_s$; (**a**) extensional stiffness, A_{11}; (**b**) coupling stiffness, B_{11}; (**c**) bending stiffness, D_{11}; (**d**) elastic modulus, $E_{xx} = E_{yy}$; (**e**) shear modulus, G_{xy}; (**f**) Poisson's ratio, ν_{xy}. The volume fraction of CF is 56%.

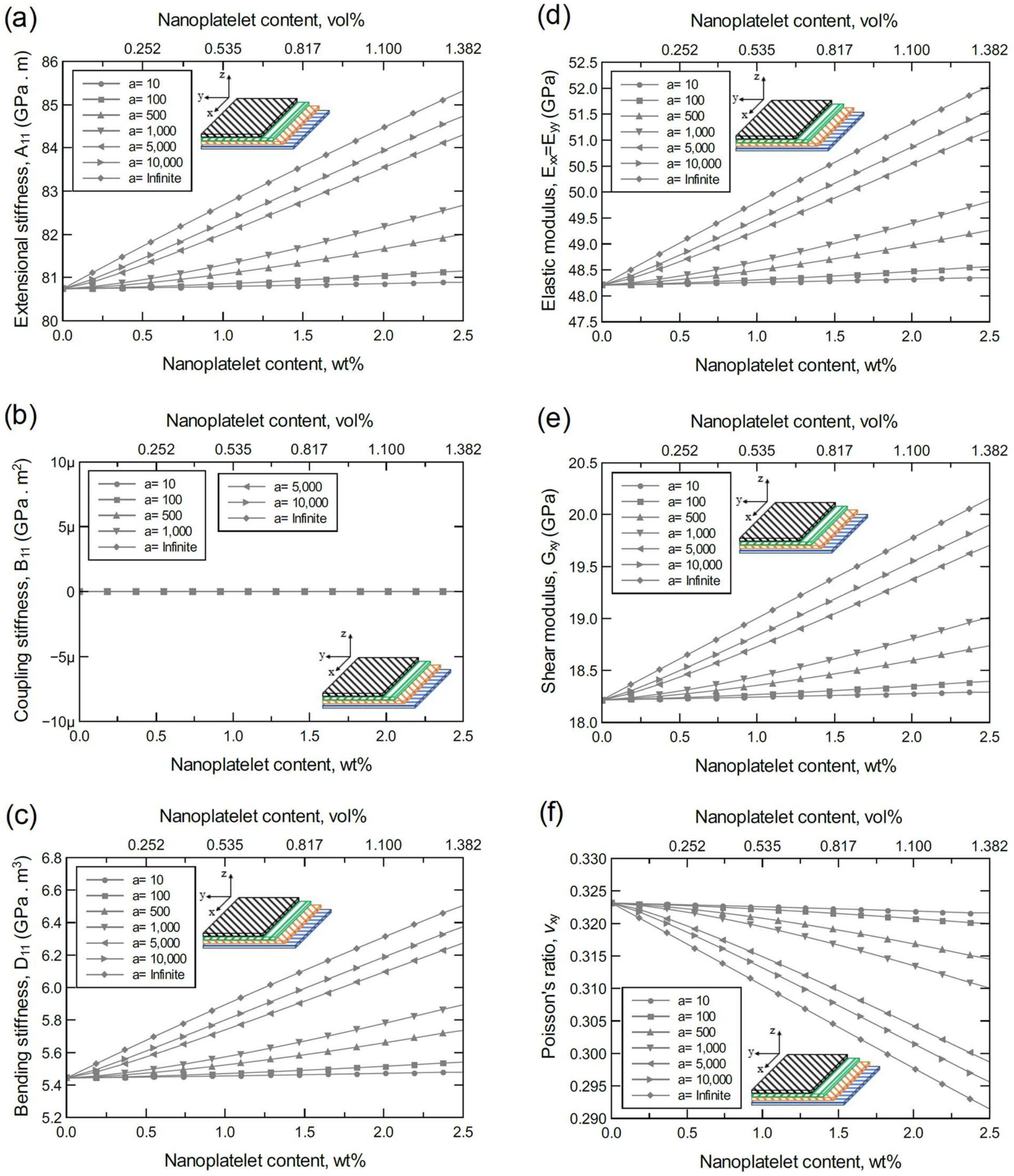

Figure S10. Predicted mechanical properties of the laminated CF/4GNP/epoxy, a symmetric balanced angle-ply laminated composite plate $[60/-60/0]_s$; (**a**) extensional stiffness, A_{11}; (**b**) coupling stiffness, B_{11}; (**c**) bending stiffness, D_{11}; (**d**) elastic modulus, $E_{xx} = E_{yy}$; (**e**) shear modulus, G_{xy}; (**f**) Poisson's ratio, ν_{xy}. The volume fraction of CF is 56%.

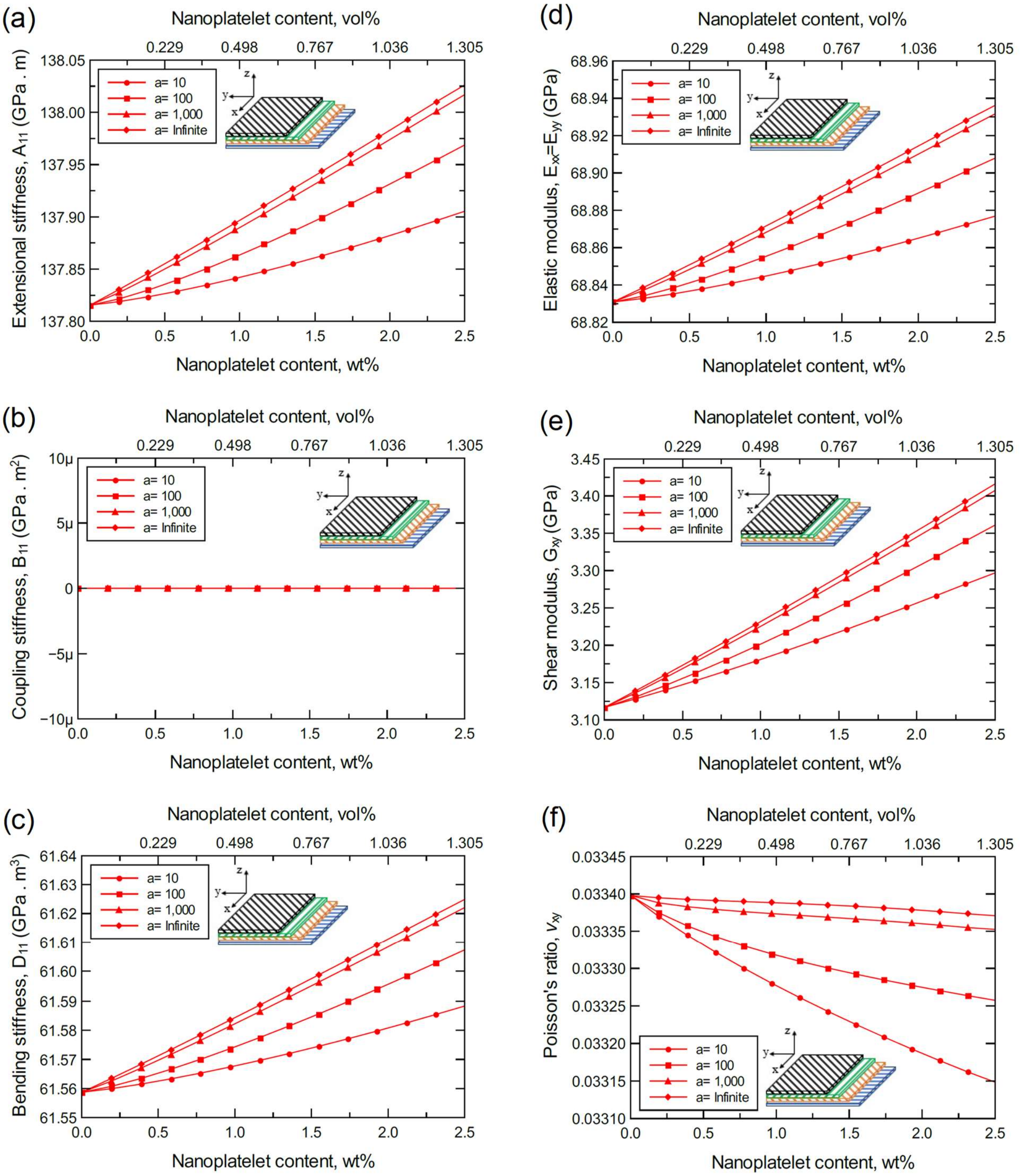

Figure S11. Predicted mechanical properties of the laminated CF/GO/epoxy, a symmetric balanced cross-ply laminated composite plate $[0/90/0/90]_s$; (**a**) extensional stiffness, A_{11}; (**b**) coupling stiffness, B_{11}; (**c**) bending stiffness, D_{11}; (**d**) elastic modulus, $E_{xx} = E_{yy}$; (**e**) shear modulus, G_{xy}; (**f**) Poisson's ratio, ν_{xy}. The volume fraction of CF is 56%.

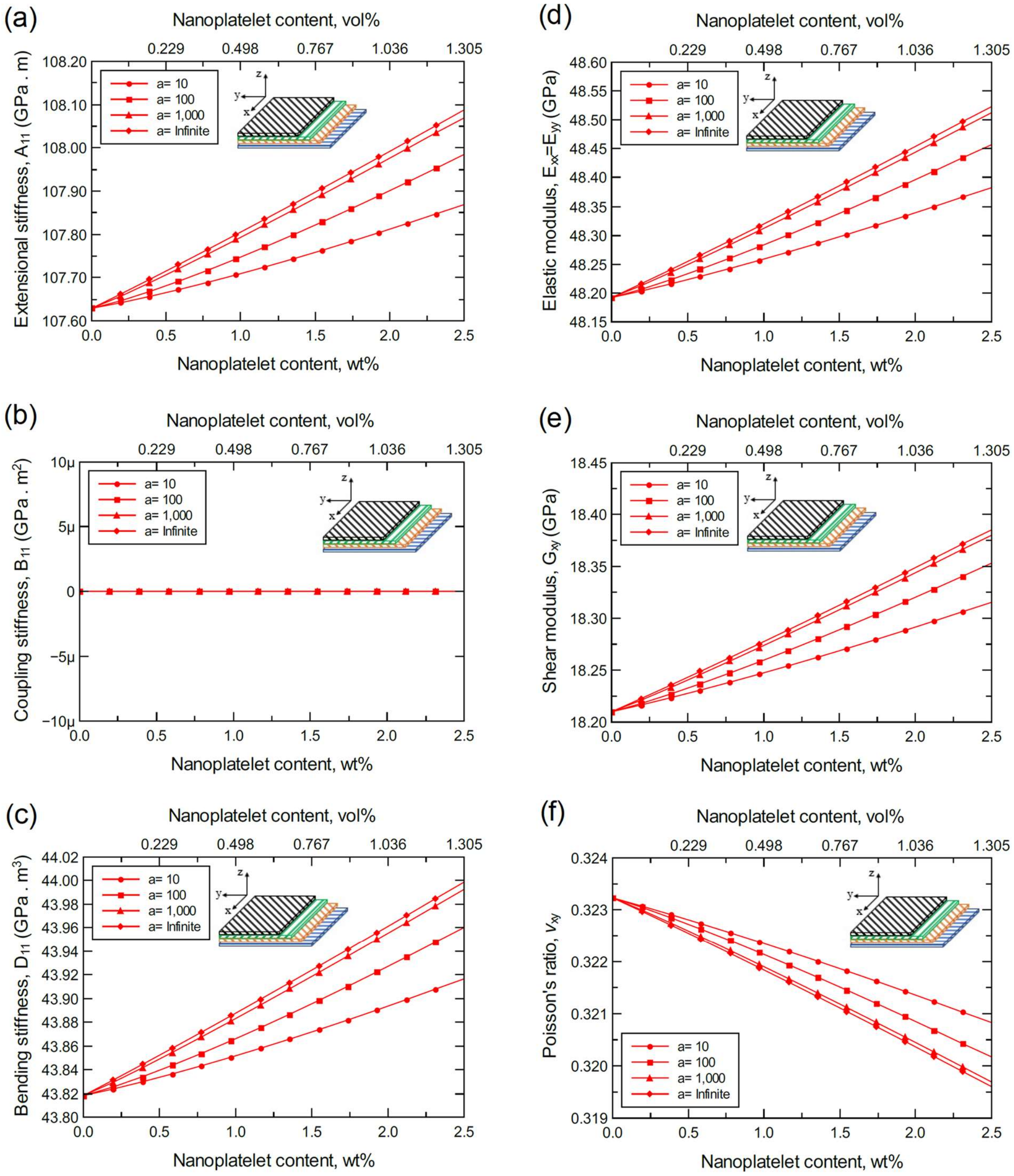

Figure S12. Predicted mechanical properties of the laminated CF/GO/epoxy, a symmetric balanced angle-ply laminated composite plate [45/0/−45/90]$_s$; (**a**) extensional stiffness, A_{11}; (**b**) coupling stiffness, B_{11}; (**c**) bending stiffness, D_{11}; (**d**) elastic modulus, $E_{xx} = E_{yy}$; (**e**) shear modulus, G_{xy}; (**f**) Poisson's ratio, ν_{xy}. The volume fraction of CF is 56%.

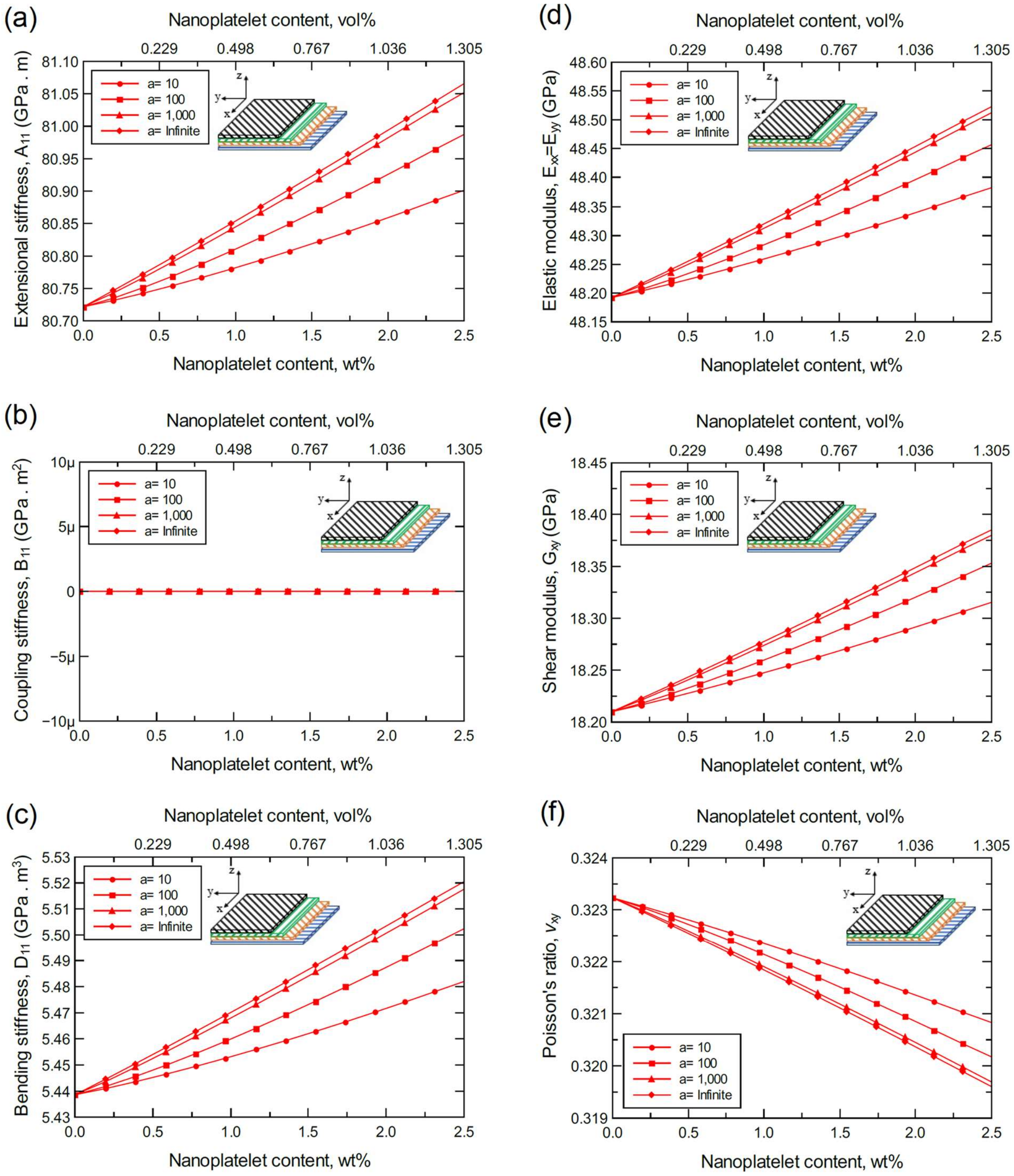

Figure S13. Predicted mechanical properties of the laminated CF/GO/epoxy, a symmetric balanced angle-ply laminated composite plate $[60/-60/0]_s$; (**a**) extensional stiffness, A_{11}; (**b**) coupling stiffness, B_{11}; (**c**) bending stiffness, D_{11}; (**d**) elastic modulus, $E_{xx} = E_{yy}$; (**e**) shear modulus, G_{xy}; (**f**) Poisson's ratio, ν_{xy}. The volume fraction of CF is 56%.

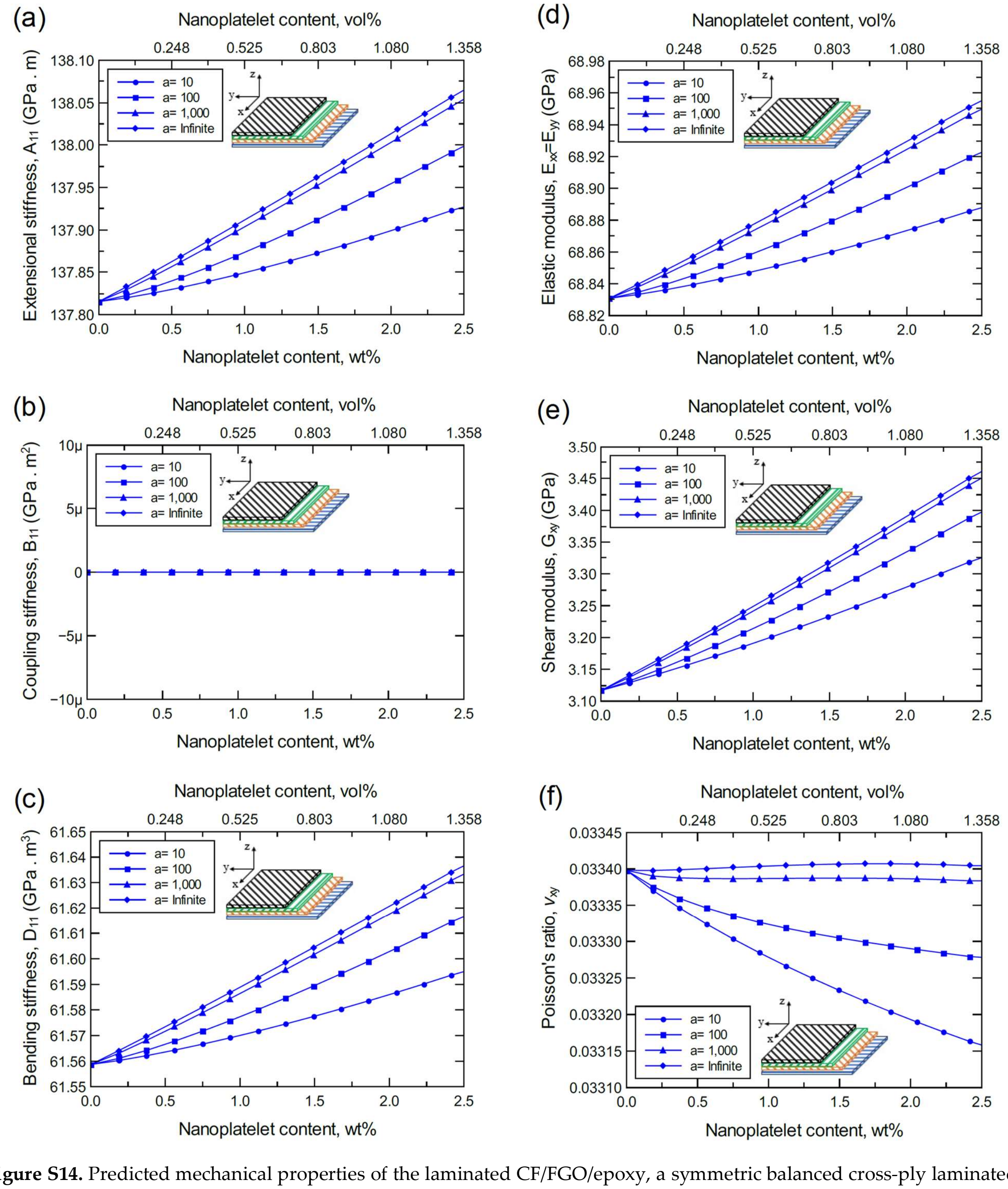

Figure S14. Predicted mechanical properties of the laminated CF/FGO/epoxy, a symmetric balanced cross-ply laminated composite plate $[0/90/0/90]_s$; (**a**) extensional stiffness, A_{11}; (**b**) coupling stiffness, B_{11}; (**c**) bending stiffness, D_{11}; (**d**) elastic modulus, $E_{xx} = E_{yy}$; (**e**) shear modulus, G_{xy}; (**f**) Poisson's ratio, ν_{xy}. The volume fraction of CF is 56%.

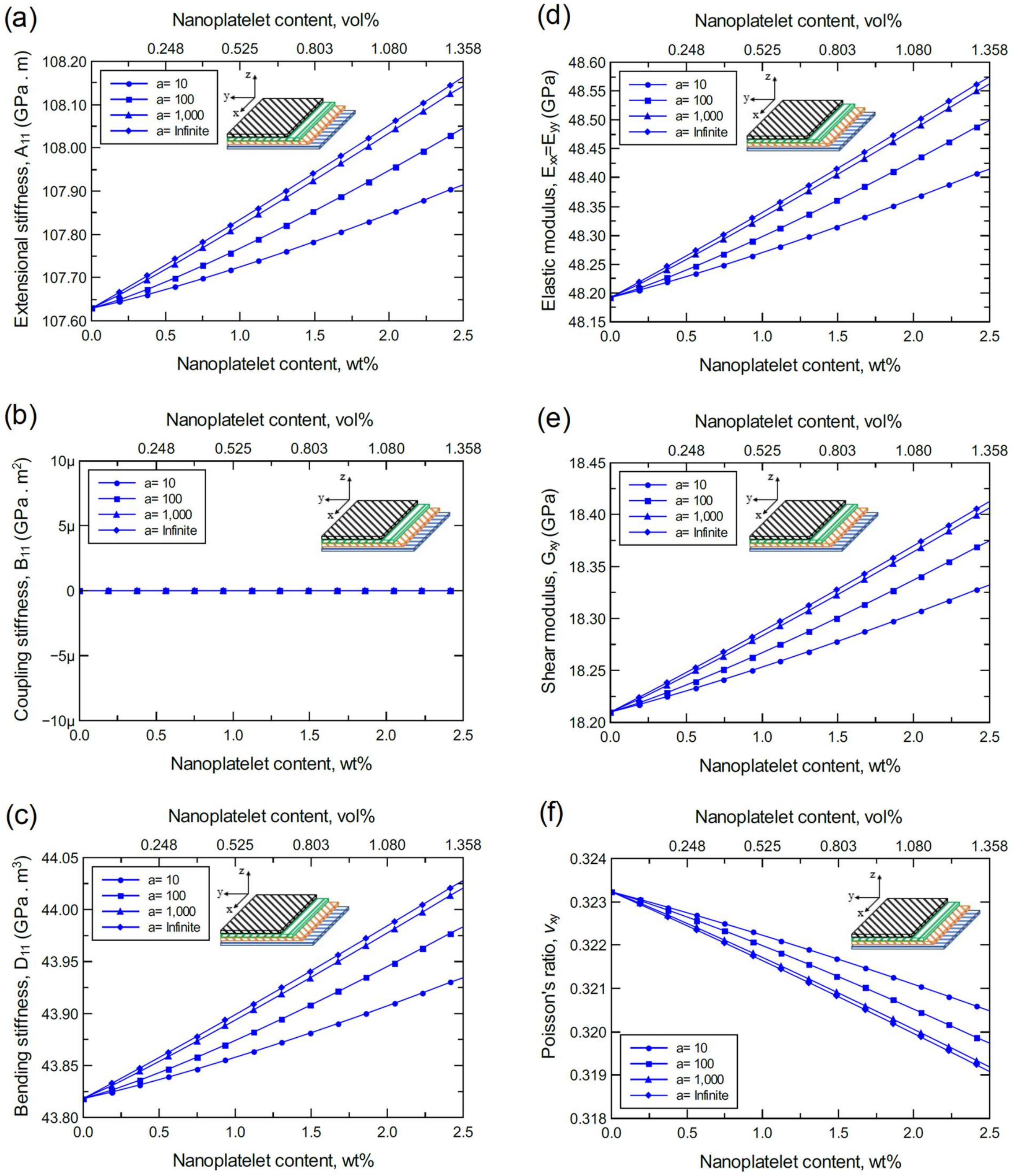

Figure S15. Predicted mechanical properties of the laminated CF/FGO/epoxy, a symmetric balanced angle-ply laminated composite plate [45/0/−45/90]$_s$; (**a**) extensional stiffness, A_{11}; (**b**) coupling stiffness, B_{11}; (**c**) bending stiffness, D_{11}; (**d**) elastic modulus, $E_{xx} = E_{yy}$; (**e**) shear modulus, G_{xy}; (**f**) Poisson's ratio, ν_{xy}. The volume fraction of CF is 56%.

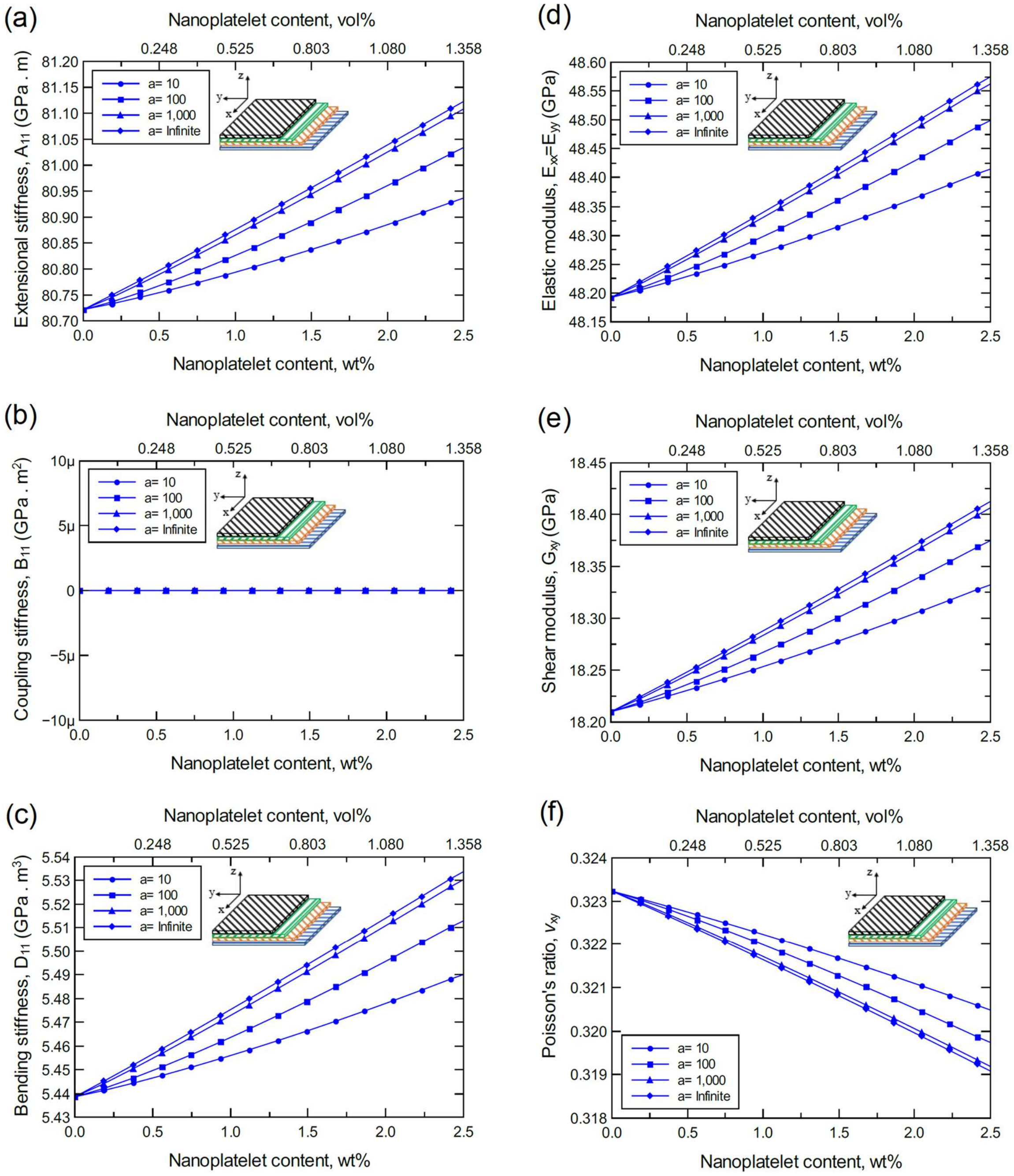

Figure S16. Predicted mechanical properties of the laminated CF/FGO/epoxy, a symmetric balanced angle-ply laminated composite plate $[60/-60/0]_s$; (**a**) extensional stiffness, A_{11}; (**b**) coupling stiffness, B_{11}; (**c**) bending stiffness, D_{11}; (**d**) elastic modulus, $E_{xx} = E_{yy}$; (**e**) shear modulus, G_{xy}; (**f**) Poisson's ratio, ν_{xy}. The volume fraction of CF is 56%.

Comparison of properties and laminate lay-ups

Figures S17–S21 show the predicted mechanical properties of the laminated hybrid composite structures with an aspect ratio of 100 for a range of nanoplatelet contents. Each plot compares the predicted elastic mechanical properties from the three laminates using the different nanoplatelet types. The plots of the normalized elastic properties compare the reinforcing effect of the nanoplatelet content on the mechanical response of the three laminates.

Considering Figure S17, the predicted extensional stiffness (A_{11}) for the cross-ply composite plate model (CP−8) is much higher than those predicted for angle-ply composite plate models (AP-8 and AP-6). As AP-8 exhibits intermediate values of A_{11}, the AP-6 exhibits the lowest A_{11} values. Nevertheless, the normalized A_{11} plots refer to an identical and larger reinforcing effect of nanoplatelet content in AP-8 and AP-6 relative to CP-8. A similar trend in the improvement of the predicted response of A_{11} can be observed with increasing the content of each of the four nanoplatelet types.

Considering Figure S18, the predicted bending stiffness (D_{11}) for the angle-ply composite plate model (AP-6) is much lower than those predicted for cross-ply composite plate models (CP-8). The D_{11} values of AP-8 are also lower, yet closer, to the D_{11} of CP-8. However, the normalized D_{11} plots refer to a larger reinforcing effect of nanoplatelet content in AP-6 relative CP-8. Furthermore, the reinforcing effect of nanoplatelet content in AP-8 is slightly higher than that in CP-8. The improvement in D_{11} involved a similar trend in all plots with increasing the content of each of the four nanoplatelet types.

Considering Figure S19, the predicted in-plane elastic modulus ($E_{xx} = E_{yy}$) of CP-8 is higher relative to that of AP-8 and AP-6. Interestingly, both AP-6 and AP-8 show an identical in-plane elastic modulus despite the difference in the number of plies. They also exhibit a better reinforcing effect with increasing the nanoplatelet content relative to that observed in CP-8. The improvement in the in-plane elastic modulus involved a similar trend in all plots with increasing the content of each of the four nanoplatelet types.

For the predicted in-plane shear modulus (G_{xy}) shown in Figure S20, opposite trends are observed relative to the predicted in-plane elastic modulus. That is, G_{xy} of CP-8 is much lower than that observed for AP-8 and AP-6 which are showing identical G_{xy} values. However, the reinforcing effect of the nanoplatelet content observed in the CP-8 is higher relative to that observed in AP-6 or AP-8. The improvement in the in-plane shear modulus involved a similar trend in all plots with increasing content of each of the four nanoplatelet types.

The predicted in-plane Poisson's ratio values (ν_{xy}) for the laminated composite plates are shown in Figure S21. The CP-8 indicates a very low lateral contraction in comparison to that observed for both AP-6 and AP-8. The normalized plots show an increase in the lateral contraction for the CP-8 system with increasing nanoplatelet content of GNP and 4GNP. Yet, the ν_{xy} slightly decreases when increasing the content of GO and FGO nanoplatelets. Also, both AP-6 and AP-8 show a decrease in the lateral contraction with increasing the nanoplatelets content. The change in the in-plane Poisson's ratio involved similar trends in all plots with increasing content of each of the four nanoplatelet types.

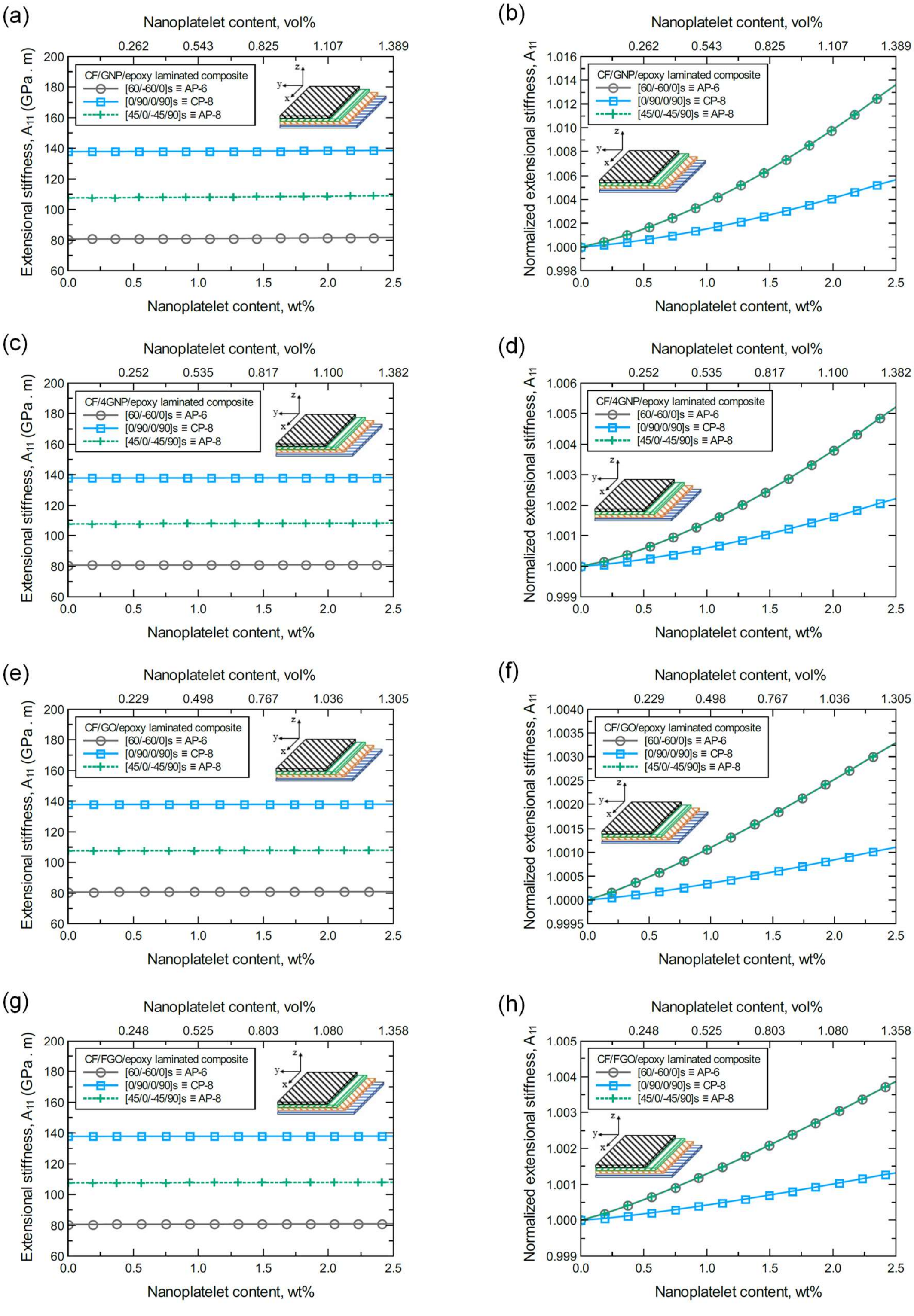

Figure S17. The predicted extensional stiffnesses (A_{11}) and normalized values for laminated hybrid composites with 100 aspect ratio and various nanoplatelet contents; (**a**) A_{11} of CF/GNP/epoxy laminated hybrid composite and its normalized values (**b**); (**c**) A_{11} of CF/4GNP/epoxy laminated hybrid composite and its normalized values (**d**); (**e**) A_{11} of CF/GO/epoxy laminated hybrid composite and its normalized values (**f**); (**g**) A_{11} of CF/FGO/epoxy laminated hybrid composite and its normalized values (**h**). The volume fraction of CF is 56%.

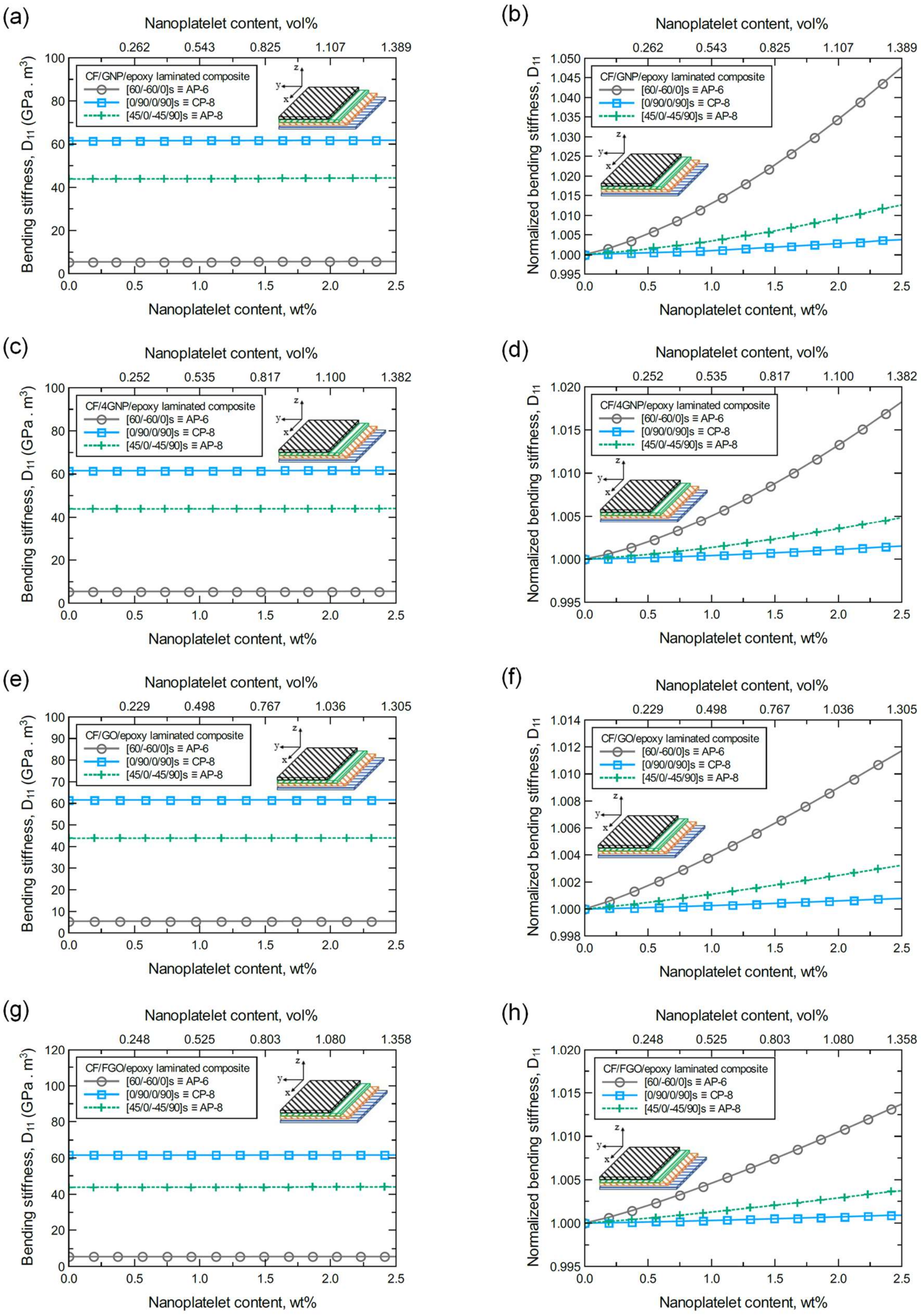

Figure S18. The predicted bending stiffnesses (D_{11}) and normalized values for laminated hybrid composites with 100 aspect ratio and various nanoplatelet contents; (**a**) D_{11} of CF/GNP/epoxy laminated hybrid composite and its normalized values (**b**); (**c**) D_{11} of CF/4GNP/epoxy laminated hybrid composite and its normalized values (**d**); (**e**) D_{11} of CF/GO/epoxy laminated hybrid composite and its normalized values (**f**); (**g**) D_{11} of CF/FGO/epoxy laminated hybrid composite and its normalized values (**h**). The volume fraction of CF is 56%.

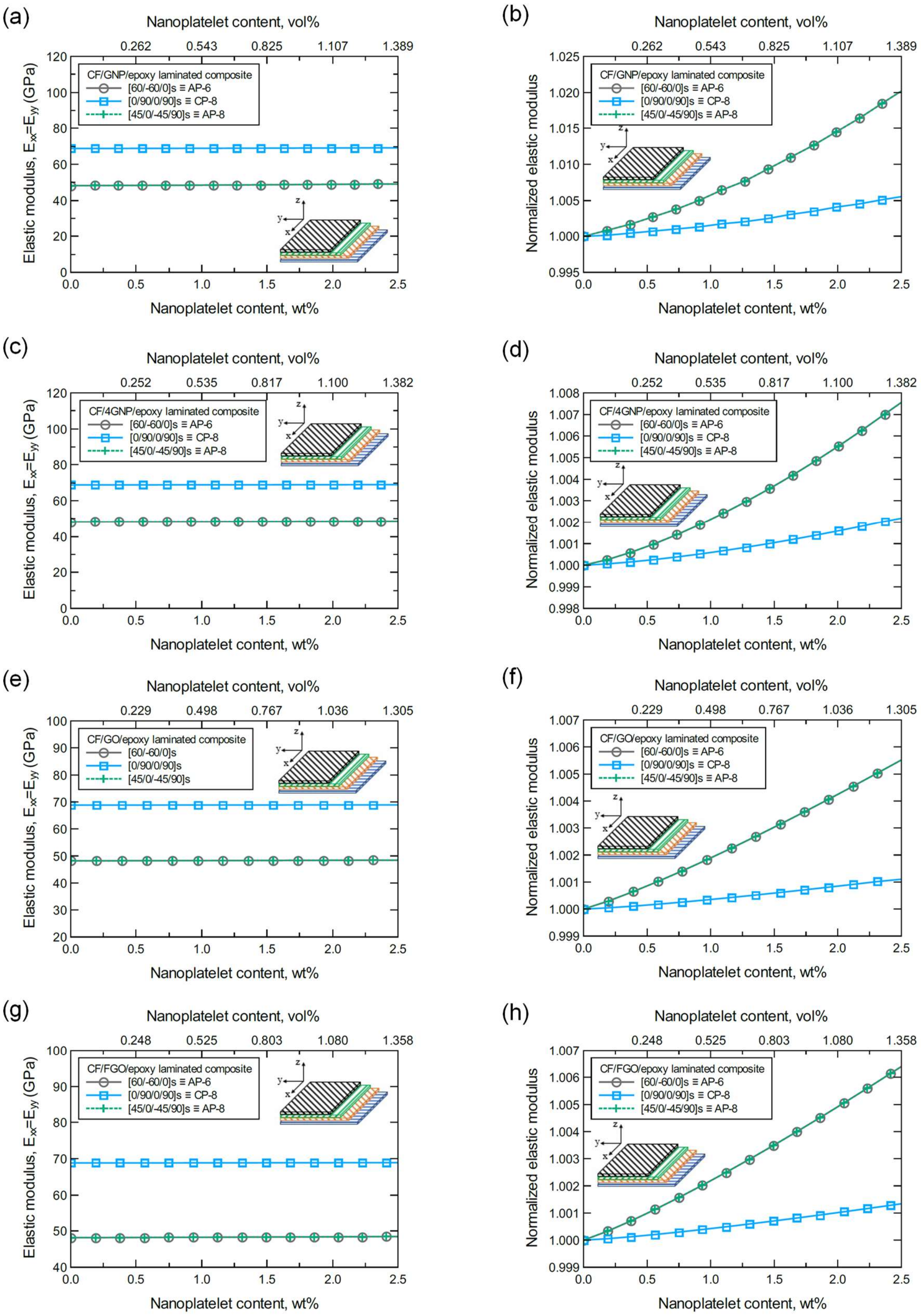

Figure S19. The predicted elastic modulus (E_{xx}= E_{yy}) and normalized values for laminated hybrid composites with 100 aspect ratio and various nanoplatelet contents; (**a**) E_{xx} of CF/GNP/epoxy laminated hybrid composite and its normalized values (**b**); (**c**) E_{xx} of CF/4GNP/epoxy laminated hybrid composite and its normalized values (**d**); (**e**) E_{xx} of CF/GO/epoxy laminated hybrid composite and its normalized values (**f**); (**g**) E_{xx} of CF/FGO/epoxy laminated hybrid composite and its normalized values (**h**). The volume fraction of CF is 56%.

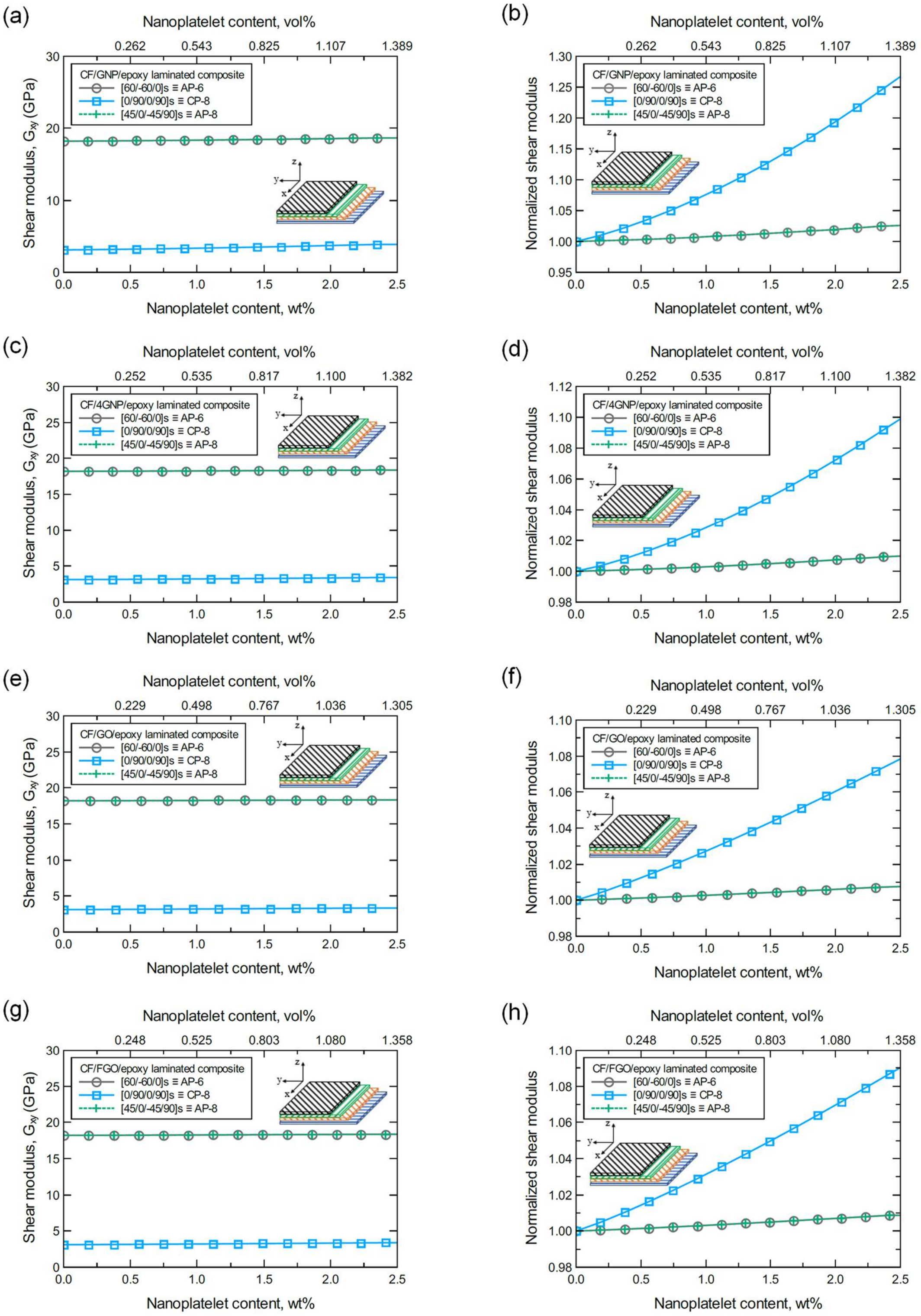

Figure S20. The predicted shear modulus (G_{xy}) and normalized values for laminated hybrid composites with 100 aspect ratio and various nanoplatelet contents; (**a**) G_{xy} of CF/GNP/epoxy laminated hybrid composite and its normalized values (**b**); (**c**) G_{xy} of CF/4GNP/epoxy laminated hybrid composite and its normalized values (**d**); (**e**) G_{xy} of CF/GO/epoxy laminated hybrid composite and its normalized values (**f**); (**g**) G_{xy} of CF/FGO/epoxy laminated hybrid composite and its normalized values (**h**). The volume fraction of CF is 56%.

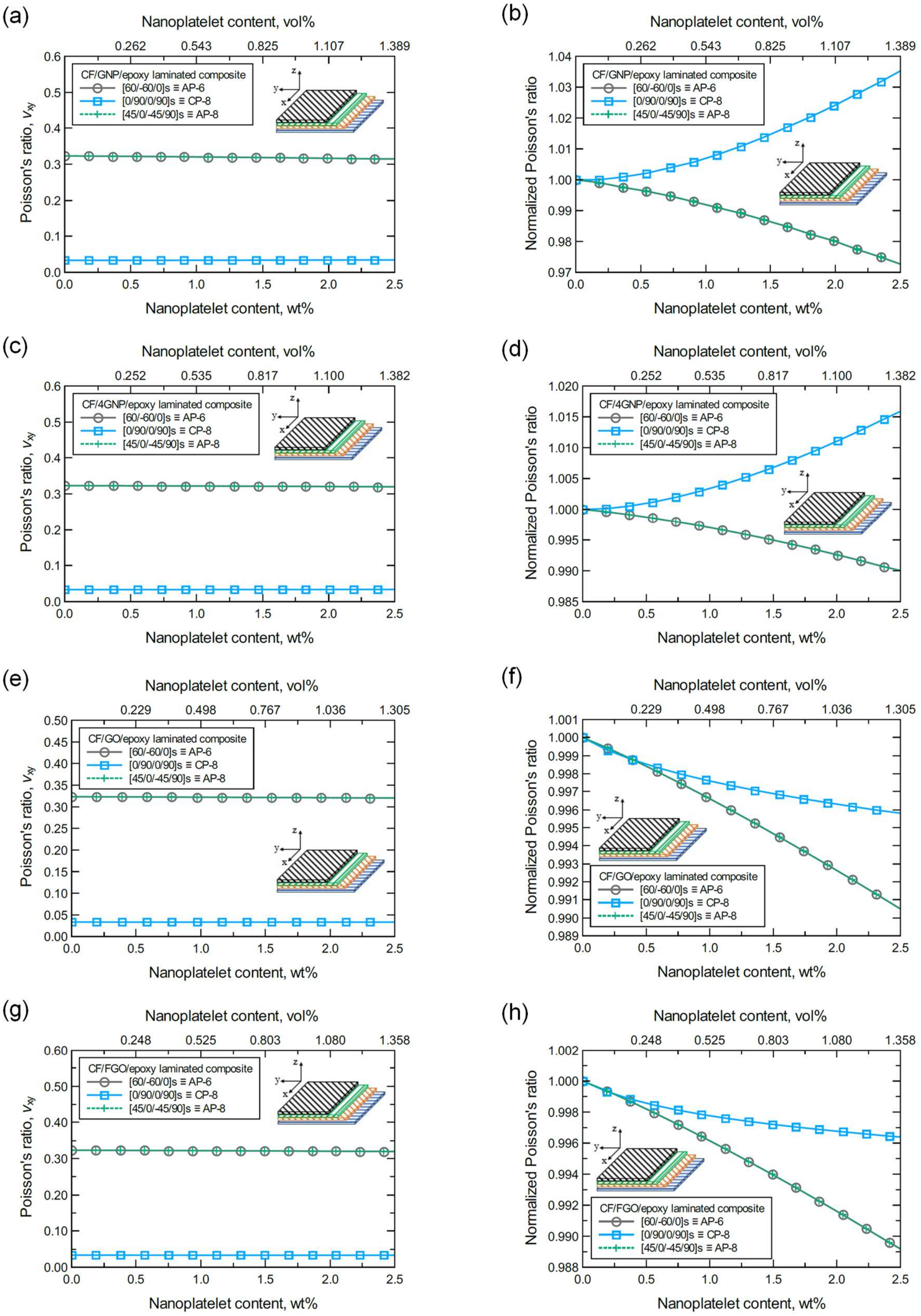

Figure S21. The predicted Poisson's ratio (ν_{xy}) and normalized values for laminated hybrid composites with 100 aspect ratio and various nanoplatelet contents; (**a**) ν_{xy} of CF/GNP/epoxy laminated hybrid composite and its normalized values (**b**); (**c**) ν_{xy} of CF/4GNP/epoxy laminated hybrid composite and its normalized values (**d**); (**e**) ν_{xy} of CF/GO/epoxy laminated hybrid composite and its normalized values (**f**); (**g**) ν_{xy} of CF/FGO/epoxy laminated hybrid composite and its normalized values (**h**). The volume fraction of CF is 56%.

References

1. Al Mahmud, H.; Radue, M.S.; Chinkanjanarot, S.; Odegard, G.M. Multiscale Modeling of Epoxy-Based Nanocomposites Reinforced with Functionalized and Non-Functionalized Graphene Nanoplatelets. *Polymers* **2021**, *13*, 1958, https://doi.org/10.3390/polym13121958.
